# Combustion Activation Induced Solid‐State Synthesis for N, B Co‐Doped Carbon/Zinc Borate Anode with a Boosting of Sodium Storage Performance

**DOI:** 10.1002/advs.202207751

**Published:** 2023-03-20

**Authors:** Hao Zhang, Dingyue Zhang, Mingyi Guo, Zheng Huang, Xu Wang, Caiqin Gao, Fan Gao, Mauricio Terrones, Yanqing Wang

**Affiliations:** ^1^ College of Polymer Science and Engineering Sichuan University Chengdu 610065 P. R. China; ^2^ Department of Physics Department of Chemistry Department of Materials Science and Engineering and Center for 2‐Dimensional and Layered Materials The Pennsylvania State University University Park PA 16802 USA

**Keywords:** borate anode, combustion activation, energy storage mechanism, ether electrolyte, sodium ion batteries

## Abstract

Zinc borates have merits of low voltage polarization and suitable redox potential, but usually suffer from low rate capability and poor cycling life, as an emerging anode candidate for Na^+^ storage. Here, a new intercalator‐guided synthesis strategy is reported to simultaneously improve rate capability and stabilize cycling life of N, B co‐doped carbon/zinc borates (CBZG). The strategy relies on a uniform dispersion of precursors and simultaneously stimulated combustion activation and solid‐state reactions capable of scalable preparation. The Na^+^ storage mechanism of CBZG is studied: 1) ex situ XRD and XPS demonstrate two‐step reaction sequence of Na^+^ storage: Zn_6_O(OH)(BO_3_)_3_+Na^+^+e^−^↔3ZnO+Zn_3_B_2_O_6_+NaBO_2_+0.5H_2_ ①, Zn_3_B_2_O_6_+6Na^+^+6e^−^↔3Zn+3Na_2_O+B_2_O_3_ ②; reaction ① is irreversible in ether‐based electrolyte while reversible in ester‐based electrolyte. 2) Electrochemical kinetics reveal that ether‐based electrolyte possesses faster Na^+^ storage than ester‐based electrolyte. The composite demonstrates an excellent capacity of 437.4 mAh g^−1^ in a half‐cell, together with application potential in full cells (discharge capacity of 440.1 mAh g^−1^ and stable cycle performance of 2000 cycles at 5 A g^−1^). These studies will undoubtedly provide an avenue for developing novel synthetic methods of carbon‐based borates and give new insights into the mechanism of Na^+^ storage in ether‐based electrolyte for the desirable sodium storage.

## Graphical abstract

N, B co‐doped carbon/zinc borates (CBZG) structure is simply prepared by an intercalator‐guided synthesis strategy as anode for sodium ion battery (SIB), showing different Na^+^ storage mechanism in ether‐ and ester‐based electrolytes and superior electrochemical performance in an ether‐electrolyte based half‐cell (437.4 mAh g^−1^ at 0.05 A g^−1^). Moreover, a Na_3_V_2_(PO_4_)_3_//CBZG full‐cell displays a high energy density of 141 Wh kg^−1^.

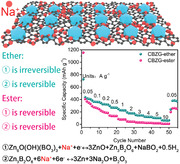

## 1 Introduction

Sodium‐ion battery (SIB) has become one of the “Top Ten Emerging Technologies in Chemistry” in 2022 from IUPAC magazine *Chemistry International*, which indicates that SIB is becoming a promising substitute for the existing lithium‐ion battery (LIB), since the economic analysis has predicted the low‐cost merit of SIB, especially when aluminum replaces copper as current collectors, cobalt and lithium minerals are scarce.^[^ ^1^, ^2^, ^3^ ^]^ Although, solvated Na^+^ possesses higher ionic conductivity than that of solvated Li^+^, a reduction potential of Li^+^ (−3.04 V vs SHE (standard hydrogen electrode)) is lower than that of Na^+^ (−2.71 V vs SHE), indicating that SIB has inferior voltage range and energy density.^[^ ^4^, ^5^, ^6^ ^]^ Besides, the huger volume variation coupling with sluggish Na^+^ diffusion in anode materials exists in SIB other than LIB owing to the higher radius of Na^+^ (Na^+^: 0.102 nm vs Li^+^: 0.076 nm).^[^ ^3^, ^5^, ^6^ ^]^ Moreover, the cathode materials of SIB—polyanionic materials, layered transition metal oxides, and Prussian blue—need a proper anode material to match their high working potential, which is a challenge.^[^ ^7^, ^8^ ^]^ Three kinds of anode materials have been explored in recent years to optimize the Na^+^ storage performance: 1) insertion‐type (graphite, hard carbon, titanium dioxide, etc.); 2) conversion‐type (Fe_2_O_3_, MoS_2_, NiP_2_, etc.); 3) alloying‐type (Sn, Bi, Sb, etc.).^[^ ^8^, ^9^, ^10^, ^11^ ^]^ Due to the different energy storage mechanisms, the insertion‐type anodes confront a limited specific capacity (300 mAh g^−1^) and the alloying‐type anodes suffer a large volume expansion (≈305–520%).^[^ ^11^, ^12^ ^]^ Compared with the above two anodes, the conversion type anodes possess advantages of abundant resources, higher theoretical capacity (≈500–1000 mAh g^−1^) and lower volume expansion (>150%), which are suitable for application in SIB.^[^ ^11^, ^13^ ^]^

Among conversion‐type anodes for SIB, transition metal borates are emergent materials with great potential application because of their low‐cost and eco‐friendly properties, low voltage polarization, and suitable redox potential.^[^ ^14^, ^15^ ^]^ In addition, the boron atom bonding with oxygen atom can form anions (such as BO_3_^3−^, BO_4_^5−^, and BO_6_^9−^) with electronegativity sites that can bond with transition metal cations and turn transition metal borates into diverse structure.^[^ ^14^, ^16^ ^]^ Therefore, transition metal borates, such as Fe_3_BO_6_,^[^ ^17^ ^]^ Fe_3_BO_5_,^[^ ^14^, ^18^, ^19^ ^]^ FeBO_3_,^[^ ^20^ ^]^ FeVBO_4_,^[^ ^16^ ^]^ Ni_2_FeBO_5_,^[^ ^21^ ^]^ VBO_3_,^[^ ^22^ ^]^ Ni_3_(BO_3_)_2_,^[^ ^23^ ^]^ Co_2_B_2_O_5_,^[^ ^24^ ^]^ and Zn_3_B_2_O_6_,^[^ ^15^, ^25^ ^]^ have been reported in SIB anodes since 2017. Their preparations are still completed by the complicated sol–gel method or undesirable grinding and mixing. Although some of the abovementioned transition metal borates demonstrated superior initial Coulombic efficiency (ICE) than that of the corresponding transition metal oxides, they showed unsatisfactory rate capability and cycling life of SIBs in carbonate ester‐based electrolytes. For example, in ester‐based electrolyte, “N‐doped Zn_3_B_2_O_6_” SIB anode offered an initial discharge capacity of 327.7 mAh g^−1^ and charge capacity of 96.4 mAh g^−1^ with an inferior ICE of 29.4%^[^ ^25^ ^]^; the other “N‐doped C@Zn_3_B_2_O_6_” SIB anode improves the ICE, but have poor rate performance (357.8 mAh g^−1^ at 0.1 A g^−1^ vs 172.8 mAh g^−1^ at 1 A g^−1^) and low cycling stability (30 cycles at 0.1 A g^−1^).^[^ ^15^ ^]^ The relatively undesirable performance in ester‐based electrolytes is attributed to the thick and dissolved solid–electrolyte–interphase (SEI) layer, sluggish de‐solvation rate, and poor compatibility in SIBs.^[^ ^26^, ^27^ ^]^ To overcome the shortcomings of ester‐based electrolyte, ether‐based electrolytes have emerged again in SIBs in recent years and show better performance in various anodes, such as graphite, hard carbon, and metal sulfide.^[^ ^8^, ^27^, ^28^ ^]^ Compared with ester‐based electrolyte, ether‐based electrolytes provide high‐quality thinner SEI layer formed on electrode surface, faster de‐solvation rate, and better compatibility with electrodes, due to the stable chemical stability and lower de‐solvation energy of ether solvent.^[^ ^26^, ^27^, ^28^, ^29^, ^30^ ^]^ Therefore, ether‐based electrolytes enable a fast Na^+^ storage kinetics and highly reversible sodiation/desodiation reaction, possessing good prospects for development in SIBs.

In this work, for the first time, we put forward an intercalator‐guided combustion activation based solid‐state synthesis strategy to construct N, B co‐doped carbon/zinc borates (labeled as CBZG, and zinc borates are Zn_6_O(OH)(BO_3_)_3_ and Zn_4_O(BO_2_)_6_); N, B co‐doped carbon/Fe_2_O_3_ (labeled as CBFG) and N, B co‐doped carbon/Co/Co_3_O_4_ (labeled as CBCG) counterparts. Compared with the transition metal borates reported in previous literature, two vital stages are concluded: 1) benefiting from the intercalator role of boric acid, the carbon precursors are self‐assembled into a 2D‐layered structure; 2) the combustion activation and solid‐state synthesis are completed in one step at proper temperature. In the latter, the concept of “combustion activation” is proposed on basis of combustion synthesis (to prepare zinc oxide) and the reaction between its gas products and carbon component, which can endow the obtained materials with more pore structure. When the as‐prepared CBZG, CBFG, and CBCG composite were used as anodes of SIB half‐cells, higher ICE, reversible capacity, and cycling performance are obtained in ether‐based electrolyte than ester‐based electrolyte. In addition, three full‐cells were assembled with pre‐sodiation CBZG, CBFG, and CBCG as anodes, respectively, and Na_3_V_2_(PO_4_)_3_ (NVP) as a cathode in ether‐based electrolyte. All full‐cells exhibit a high energy density of 141, 154, and 168 Wh kg^−1^, respectively, and a superior cycling life (2000 cycles at 5 A g^−1^). In order to study the distinct mechanisms of CBZG anodes in ether‐ and ester‐based SIBs, ex situ X‐ray diffraction (XRD) and X‐ray photoelectron spectroscopy (XPS) were employed. The possible sodium‐ion storage mechanism in ether‐based electrolyte is: Zn_6_O(OH)(BO_3_)_3_ + Na^+^ + e^−^ → 3ZnO + Zn_3_B_2_O_6_ + NaBO_2_ + 0.5H_2_; Zn_3_B_2_O_6_ + 6Na^+^ + 6e^−^ ↔ 3Zn + 3Na_2_O + B_2_O_3_; in ester‐based electrolyte is: Zn_6_O(OH)(BO_3_)_3_ + Na^+^ + e^−^ ↔ 3ZnO + Zn_3_B_2_O_6_ + NaBO_2_ + 0.5H_2_; Zn_3_B_2_O_6_ + 6Na^+^ + 6e^−^ ↔ 3Zn + 3Na_2_O + B_2_O_3_. Subsequent kinetic tests showed that the Na^+^ storage kinetics in ether‐based electrolyte is faster than that in ester‐based electrolyte. These encouraging results indicate that our work opens a new synthesis method via intercalator‐guided combustion activation based solid‐state synthesis and offers new insights on sodium‐ion storage mechanisms in ether‐ and ester‐based electrolytes.

## 2 Results and Discussion

### 2.1 Synthesis, Structure, and Chemistry

**Figure 1:**
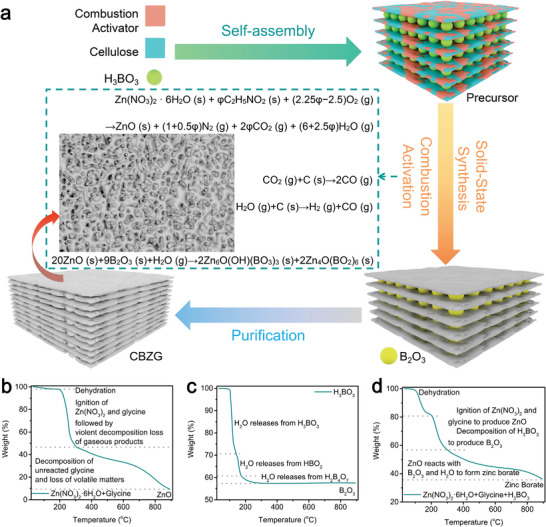
a) Illustration of the synthesis process for CBZG. TG curves of b) the mixture of Zn(NO_3_)_2_·6H_2_O and glycine, c) H_3_BO_3_, and d) the mixture of Zn(NO_3_)_2_·6H_2_O, glycine and H_3_BO_3_.

We chose hydroxypropyl cellulose as a carbon source (labeled as C), H_3_BO_3_ as an intercalator (labeled as B),^[^ ^31^ ^]^ Zn(NO_3_)_2_⋅6H_2_O as an oxidizer (labeled as Z), and glycine as a fuel (labeled as G) to prepare N, B co‐doped carbon/zinc borate (labeled as CBZG from the mentioned abbreviation of materials) by an intercalator‐guided combustion activation based solid‐state synthesis method. **Figure** **1**a illustrates three key steps of the synthesis: 1) self‐assembly of hydroxypropyl cellulose, H_3_BO_3_, Zn(NO_3_)_2_⋅6H_2_O and glycine into a 2D layer‐structured precursor by evaporative recrystallization at 90 °C, due to the formation of plate‐like H_3_BO_3_ crystals in this process; 2) pyrolysis of the above precursor at 900 °C under an inert atmosphere to complete combustion activation and solid‐state synthesis process; and 3) purification of the pyrolysis product to obtain CBZG sample (Equation 1). The intercalator‐free, oxidizer‐free, or fuel‐free samples were also fabricated for comparison in the same way (Z, CZ, CZG, BZ, and CBZ). The above synthesis method is simple and green for scalable preparation. When Fe(NO_3_)_3_⋅9H_2_O or Co(NO_3_)_2_⋅6H_2_O replaced Zn(NO_3_)_2_⋅6H_2_O, the corresponding N, B co‐doped carbon/iron borate or N, B co‐doped carbon/cobalt borate was not produced, but N, B co‐doped carbon/Fe_2_O_3_ (labeled as CBFG, Equation 2) or N, B co‐doped carbon/Co/Co_3_O_4_ (labeled as CBCG, Equation 2) was formed, which may be attributed to the non‐reaction between B_2_O_3_ and Fe_2_O_3_/Co_3_O_4_ or the insufficient reaction time. The mentioned reactions are as follows:

(1) $$B_{2}O_{3}\left(s\right)+3H_{2}O\left(l\right)\to 2H_{3}{\mathrm{BO}}_{3}\left(\mathrm{aq}\right)$$

(2) $$\begin{array}{ccc} & & M^{v}{\left({\mathrm{NO}}_{3}\right)}_{v}\left(s\right)+\left(v\varphi \right)5/9C_{2}H_{5}{\mathrm{NO}}_{2}\left(s\right)+v\left(\varphi -1\right)5/4O_{2}\left(g\right)\\ & & \;\to M^{v}O_{v/2}\left(s\right)+\left(v\varphi \right)10/9{\mathrm{CO}}_{2}\left(g\right)+\varphi 25/18H_{2}O\left(g\right)\\ & & \;\;\;\;\;+\;v\left(\varphi 5/9+1\right)/2N_{2}\left(g\right) \end{array}$$

where M*^v^* denotes a *v*‐valent metal, and *φ* = 1 implies that the nitrate and glycine mixture does not need O_2_ to completely oxidize fuel, while *φ* > 1 (<1) means fuel‐rich (lean) conditions.^[^ ^32^ ^]^

(3) $$\begin{array}{ccc} 2\mathrm{Zn}{\left({\mathrm{NO}}_{3}\right)}_{2}\cdot 6H_{2}O\left(s\right) & \to & 2\mathrm{ZnO}\left(s\right)+4{\mathrm{NO}}_{2}\left(g\right)+O_{2}\left(g\right)\\ & & +\;12H_{2}O\left(g\right) \end{array}$$

(4) $$2H_{3}{\mathrm{BO}}_{3}\left(s\right)\to B_{2}O_{3}\left(s\right)+3H_{2}O\left(g\right)$$

(5) $${\mathrm{CO}}_{2}\left(g\right)+C\left(s\right)\to 2\mathrm{CO}\left(g\right)$$

(6) $$H_{2}O\left(g\right)+C\left(s\right)\to H_{2}\left(g\right)+\mathrm{CO}\left(g\right)$$

(7) $$\begin{array}{ccc} 20\mathrm{ZnO}\left(s\right)+9B_{2}O_{3}\left(s\right)+H_{2}O\left(g\right) & \to & 2{\mathrm{Zn}}_{6}O\left(\mathrm{OH}\right){\left({\mathrm{BO}}_{3}\right)}_{3}\left(s\right)\\ & & +\;2{\mathrm{Zn}}_{4}O{\left({\mathrm{BO}}_{2}\right)}_{6}\left(s\right) \end{array}$$

The Equation 2 reflects the combustion process, which are supported by Thermogravimetric analysis (TG) analysis of Zn(NO_3_)_2_⋅6H_2_O, glycine (Figure S1, Supporting Information), and the mixture of Zn(NO_3_)_2_⋅6H_2_O and glycine (Figure 1b). The reaction temperature of Zn(NO_3_)_2_⋅6H_2_O and glycine to form ZnO ranges from 200 to 300 °C, which is lower than the decomposition temperature of Zn(NO_3_)_2_⋅6H_2_O (≈100–550 °C, Equation 3). This indicates that the combustion process decreases the formation temperature and time of ZnO, which is favor of ZnO reacting with B_2_O_3_ decomposed from H_3_BO_3_ (≈100–300 °C, Figure 1c and Equation 4) at low temperature. The CO_2_ and H_2_O formed by combustion process can react with carbon derived from hydroxypropyl cellulose to construct porous structure (activation process, Equations 5 and 6). So, combined with reactions (2, 5, and 6), we propose the concept of “combustion activation” to summarize the reaction process. In the TG curve of the mixture of Zn(NO_3_)_2_⋅6H_2_O, glycine, and H_3_BO_3_ (Figure 1d), the continuous weight‐loss consists of three stages: 1) the dehydration of Zn(NO_3_)_2_⋅6H_2_O (ca. 40–200°C), 2) the decomposition of H_3_BO_3_ as well as the ignition between Zn(NO_3_)_2_ and glycine (≈200–300°C), and 3) the reaction among ZnO, B_2_O_3_, and H_2_O to form zinc borate (Equation 7, >300°C). The roles of glycine in material synthesis process are: 1) Glycine participates in the combustion activation process further to release a large amount of gas, create pores in the carbon matrix and promote the formation of plate stacking structure. 2) Glycine serves as nitrogen dopants to complete the N doping of the resulting materials. 3) Glycine reacts with Zn(NO_3_)_2_⋅6H_2_O to reduce the consumption of carbon. In a word, we utilize the advantages of the layer‐structure of H_3_BO_3_, the combustion activation among Zn(NO_3_)_2_, glycine, and carbon, and the solid‐state synthesis among ZnO, B_2_O_3_, and H_2_O to create an intercalator‐guided combustion activation based solid‐state synthesis method, which can successfully construct CBZG material.

**Figure 2:**
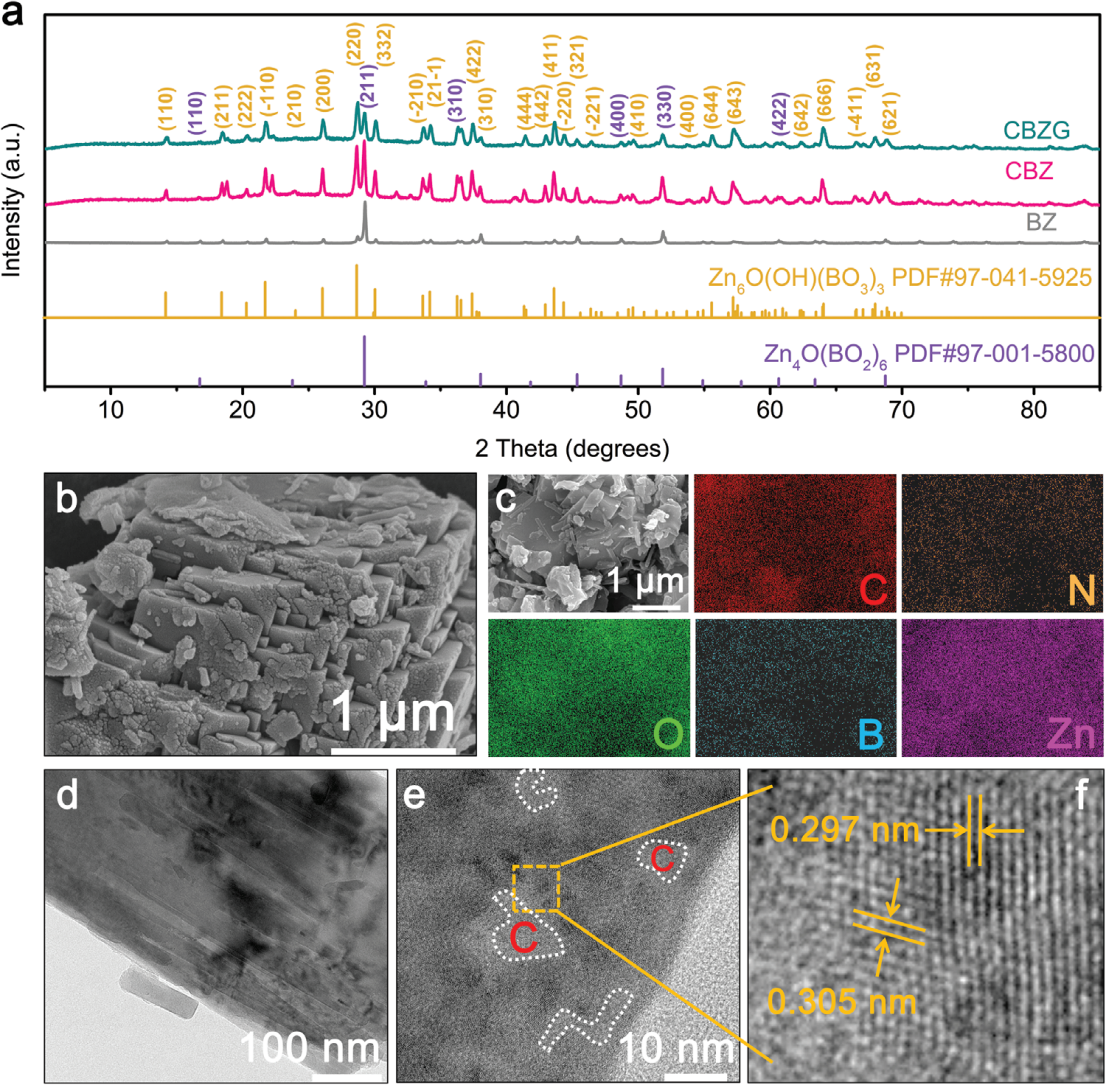
Structure and morphology of the obtained materials. a) XRD patterns of BZ, CBZ, and CBZG. b) SEM image of CBZG. c) Element mapping images of CBZG. d) TEM image of CBZG. e,f) High‐resolution TEM images of CBZG.

To identify the phase of the obtain materials, X‐ray diffraction (XRD) was used. **Figure** **2**a demonstrates two main phases of Zn_4_O(BO_2_)_6_ (PDF#97‐001‐5800) and Zn_6_O(OH)(BO_3_)_3_ (PDF#97‐041‐5925) in the XRD patterns of BZ, CBZ, and CBZG samples and the corresponding crystal structures of Zn_4_O(BO_2_)_6_ and Zn_6_O(OH)(BO_3_)_3_ are displayed in Figure S2 (Supporting Information). The relative crystalline peak intensity of Zn_6_O(OH)(BO_3_)_3_ phase increases and Zn_4_O(BO_2_)_6_ phase decreases simultaneously with the addition of hydroxypropyl cellulose or glycine (from BZ to CBZ, and then to CBZG), which may be due to the increase of carbon content by combustion activation. The theoretical capacities of Zn_6_O(OH)(BO_3_)_3_ and Zn_4_O(BO_2_)_6_ are calculated ≈579.0 and 401.2 mAh g^−1^, respectively. Without adding H_3_BO_3_, the main phase of Z, CZ, and CZG samples is ZnO (PDF#36‐1451), as displayed in the XRD patterns (Figure S2, Supporting Information). Scanning electron microscopy (SEM) and transmission electron microscopy (TEM) characterize the evolution process of sample morphology. As exhibited in the SEM images (Figure S3, Supporting Information), From Z to CZ, and to CZG, the block‐like morphology gradually changes into a particle aggregation structure (combustion activation); from BZ to CBZ, the box‐like morphology turns into a disorderly stacked plate‐like structure (solid‐state synthesis). When combining combustion activation and solid‐state synthesis, the morphology of CBZG is a block structure with clear edges and corners, which is accumulated layer by layer from plate (Figure 2b and Figure S4, Supporting Information). Moreover, C, N, O, B, and Zn elements are uniformly distributed on the CBZG, as shown in the elemental mapping images (Figure 2c). TEM images manifest the same morphological evolution trend as SEM test results (Figure 2d and Figure S5, Supporting Information). The high‐resolution TEM images of CBZG show the structure of disordered carbon surrounded by crystals of zinc borate (Figure 2e, white dotted circle, and Figure S6, Supporting Information) and the obvious crystal lattices with 0.297 nm and 0.305 nm interlayer spacings, representing the (332) crystal plane of Zn_6_O(OH)(BO_3_)_3_ phase and the (211) crystal plane of Zn_4_O(BO_2_)_6_ phase, respectively (Figure 2f, yellow mark). The carbon embedded in zinc borate crystals can increase conductivity and provide more active sites for electrochemical reaction, which is conducive to improving Na^+^ storage performance.

**Figure 3:**
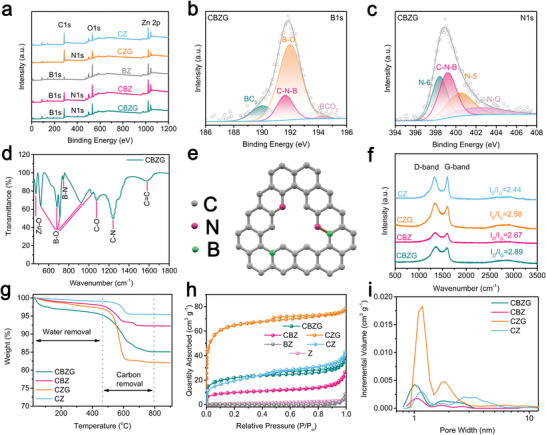
Physical and chemical properties of the obtained materials. a) XPS survey spectra of the obtained materials. High‐resolution b) B 1s and c) N 1s XPS spectra of CBZG. d) FTIR spectrum of CBZG. e) Schematic illustration of B, N co‐doped carbon matrix. f) Raman spectra of CZ, CZG, CBZ, CBZG. g) TG curves of CZ, CZG, CBZ, CBZG. h,i) N_2_ adsorption‐desorption isotherms and pore size distribution curves of various samples.

The X‐ray photoelectron spectroscopy (XPS) was employed to investigate the chemical properties of the obtain materials (**Figure** **3**a and Figure S7, Supporting Information) and the corresponding elemental composition is in Table S1 (Supporting Information). The content of N element for CBZG was calculated to be 5.01 at%, which is higher than 1.87 at% of CBZ and 2.33 at% of CZG, suggesting more N atoms can be reserved after synergistic effect of combustion activation and solid‐state synthesis. For the high‐resolution XPS B 1s spectra of CBZG (Figure 3b), besides the chemical bonds associated with BC_3_ (190.0 eV), B‐O (192.0 eV), and BCO_2_ (194.3 eV), a characteristic peak at 191.6 eV is C‐N‐B chemical bonding (the proportion is 16.40%, Table S2, Supporting Information).^[^ ^33^, ^34^ ^]^ For the high‐resolution XPS N 1s spectra of CBZG (Figure 3c), four characteristic peaks were observed, centered at 398.4, 399.2, 400.4, and 402.2 eV, corresponding to N‐6, C‐N‐B (the proportion is 33.15%, Table S2, Supporting Information), N‐5, and N‐Q, respectively.^[^ ^35^, ^36^ ^]^ For the high‐resolution XPS C 1s spectra of CBZG (Figure S7, Supporting Information), C=C/C—C (284.6 eV), C—O/C—N/C—N—B (285.8 eV), and C=O (288.7 eV) bonds were obtained after fitting.^[^ ^33^, ^35^ ^]^ The existence of C—N—B bond can also be found in CBZ (Figure S7, Supporting Information) and the proportion of C—N—B bond in B 1s and N 1s is 4.56% and 28.59%, respectively, which is lower than that of CBZG (Table S2, Supporting Information). Fourier transform infrared spectroscopy (FTIR) also proves the existence of C‐N‐B bond in CBZG (Figure 3d), which suggests the bonding of B and N in carbon matrix. Based on the above‐mentioned evidence, the possible presence status of B and N atoms in carbon framework is displayed in Figure 3e. The incorporation of B and N co‐doping can improve the energy band structure of carbon atoms and increase defects further to boost Na^+^ storage performance.

As shown in Raman spectra of Figure 3f, two main peaks center at about 1348 and 1601 cm^−1^, corresponding to the D‐band (related to sp^3^ defective carbon) and the G‐band (related to sp^2^ graphitic carbon).^[^ ^36^, ^37^ ^]^ The I_D_/I_G_ value (quantify the concentration of defects in carbon) of CZ, CZG, CBZ, and CBZG calculated from fitted Raman spectra (Figure S8, Supporting Information) is 2.44, 2.58, 2.67 and 2.89, respectively, manifesting that the increase of B, N‐doping content produce more disorder and defects. Besides Na^+^ intercalation, sodium storage mechanism in the low potential region of disordered carbon will be reversible Na^+^ adsorbed at graphene defects (di‐vacancies and Stone‐Wales) and at heteroatom functionalities, reversible Na atoms filled micropores.^[^ ^38^, ^39^ ^]^ The carbon content of CZG and CBZG is ≈14.2 and 10.1 wt.%, respectively, which is higher than that of CZ and CBZ (3.5 wt.%; 5.3 wt.%), calculated by TG under air flow (Figure 3g). Additionally, the content of Zn_4_O(BO_2_)_6_ and Zn_6_O(OH)(BO_3_)_3_ in CBZG are calculated about 42.3 and 47.6 wt.%, respectively. From the mass loss values of carbon in the obtained materials, it can be concluded that the introduction of combustion activation in the synthesis process is conducive to increasing the carbon content of the products since no oxygen was generated in combustion activation to consume carbon materials during the pyrolysis, while non‐combustion‐activation is contrary. As displayed in Figure 3h,i, all carbon containing samples show typical I/IV type N_2_ adsorption‐desorption isotherms and similar trend of pore size distribution, implying hierarchically porous structure.^[^ ^35^, ^36^, ^37^ ^]^ The Brunauer–Emmett–Teller (BET) specific surface area of CZG and CBZG is 238 m^2^ g^−1^ and 79 m^2^ g^−1^, respectively, which is larger than Z of 4 m^2^ g^−1^, CZ of 70 m^2^ g^−1^, BZ of 0.4 m^2^ g^−1^, and CBZ of 36 m^2^ g^−1^ (Table S3, Supporting Information). Furthermore, the total pore volume of Z, CZ, CZG, BZ, CBZ, and CBZG reaches 0.006, 0.055, 0.116, 0.002, 0.028, and 0.047 cm^3^ g^−1^, respectively (Table S3, Supporting Information). These values confirm that the higher carbon content the sample has, the larger BET specific surface area and total pore volume the sample obtains, which is ascribed to the introduction of combustion activation. To sum up, the hierarchically porous structure combined with high heteroatom content will offer more interfacial Na adsorption sites for contributing capacity via capacitive Na^+^ storage, buffer volume change of conversion reaction process for zinc borate, store charges below 0.1 V by Na atom underpotential deposition (micropores).^[^ ^38^, ^39^, ^40^, ^41^, ^42^ ^]^

### 2.2 Sodium Storage Performance and Mechanism

The above “Z” containing samples were used as anodes of SIB half‐cells to assess electrochemical performance in electrolyte of 1 m NaFSI in diethylene glycol dimethyl ether (DEGDME, ether). Galvanostatic charge–discharge profiles at 0.05 A g^−1^ in the range of 0.01–3.0 V (vs Na^+^/Na) and rate capability at different current densities are showed in Figure S9 (Supporting Information). The reversible capacity of CBZG anode is 484.7 mAh g^−1^, which is higher that of Z (86.2 mAh g^−1^), CZ (140.8 mAh g^−1^), CZG (191.6 mAh g^−1^), BZ (187.3 mAh g^−1^), and CBZ (404.2 mAh g^−1^). Meanwhile, CBZG anode exhibits the best capacity at each current density compared with other five anodes. The lowest charge transfer resistance (*R*_ct_, 21.9 Ω) of CBZG anode among all “Z” containing samples exhibits in Figure S9 and Table S4 (Supporting Information). When CBZG anode with a higher mass loading of ≈2.0 mg cm^−2^, it also shows a good rate and cycle performance in Figure S10 (Supporting Information). All the above results show the high sodium storage performance of CBZG anode, which can be attributed to the special structure increasing the contact area with electrolyte, the high carbon and heteroatom content improving conductivity and adsorption ability for Na^+^, the high specific surface area accommodating volume variation of conversion reaction process, and the different conversion mechanisms between zinc borate and ZnO.

**Figure 4:**
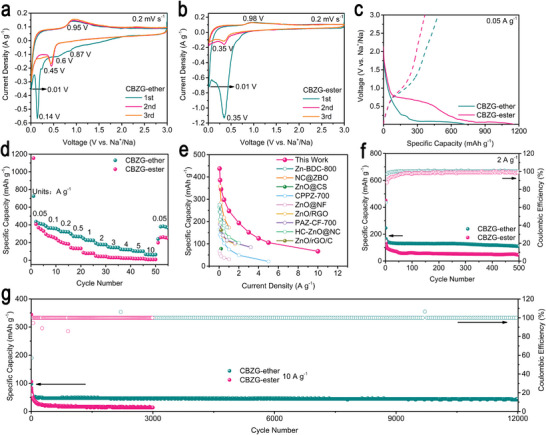
Na^+^ storage performance of CBZG as the half‐cell anode in ether‐ or ester‐based electrolyte. a,b) CV curves at 0.2 mV s^−1^. c) Discharge/charge profiles at 0.05 A g^−1^. d) Rate capability. e) Comparison between CBZG anode and previously reported SIB anodes in capacity. Cycling performance at f) 2 A g^−1^ and g) 10 A g^−1^.

To investigate why CBZG anode has such good performance, we studied the performance of SIB half‐cells in two electrolytes of 1 m NaFSI in DEGDME and 1 m NaFSI in ethylene carbonate and diethyl carbonate (EC: DEC = 1:1 in volume, ester). **Figure** **4**a, b display the initial three Cyclic voltammetry (CV) curves of CBZG anode at 0.2 mV s^−1^ in ether‐ or ester‐based electrolyte. For ether‐based electrolyte, four cathodic peaks are found at 0.87, 0.6, 0.14, and 0.01 V in the first cycle, which are different from that of ester‐based electrolyte at 0.35 and 0.01 V. In both electrolytes, the cathodic peak near 0.01 V correlate to the Na^+^ intercalation into carbon interlayers or the pore filling; the cathodic peak above 0.01 V correspond to the irreversible Na^+^ trapped in carbon, the formation of SEI layer, and the conversion reaction of zinc borate.^[^ ^38^, ^39^, ^43^ ^]^ The first cycle CV curve area of ester‐based electrolyte is larger than that of ether‐based electrolyte, thus delivering lower initial Coulombic efficiency (ICE). However, the subsequent CV curves in both electrolytes are almost overlapped, indicating the good structural stability of the CBZG anode.

Galvanostatic charge–discharge profiles of CBZG anode at various current densities in ether‐ or ester‐based electrolyte are showed in Figure 4c and Figure S9 (Supporting Information). The ICE at 0.05 A g^−1^ of ester‐based electrolyte is 31.7%, which is much lower than that of ether‐based electrolyte (66.8%). Meanwhile, as exhibited in Figure 4d, the ether‐based electrolyte possesses better rate capability of 437.4, 385.7, 345.0, 298.0, 247.6, 193.4, 151.7, 124.5, 105.6, 66.7, and 384.5 mAh g^−1^ at 0.05, 0.1, 0.2, 0.5, 1, 2, 3, 4, 5, 10, and 0.05 A g^−1^, than ester‐based electrolyte (408.0, 294.5, 221.2, 142.6, 86.2, 47.8, 33.4, 25.6, 19.5, 11.1, and 261.6 mAh g^−1^ at the same current densities). The capacities of ester‐based electrolyte above 0.2 A g^−1^ drop sharply while that of ether‐based electrolyte keep stable, indicating ether‐based electrolyte has outstanding kinetics at high current densities. When compared with previous reported zinc borate or ZnO based anodes, CBZG anode is still competitive and superior (Figure 4e).^[^ ^15^, ^25^, ^44^, ^45^, ^46^, ^47^, ^48^, ^49^, ^50^ ^]^ The cycling performance of CBZG anode in ether‐ or ester‐based electrolyte is also displayed in Figure 4f,g. In contrast to ester‐based electrolyte, ether‐based electrolyte has a higher capacity retention of 80.0% after 500 cycles at 2 A g^−1^, a larger capacity retention of 84.3% and nearly 100% columbic efficiency, even after 12 000 cycles at 10 A g^−1^.

**Figure 5:**
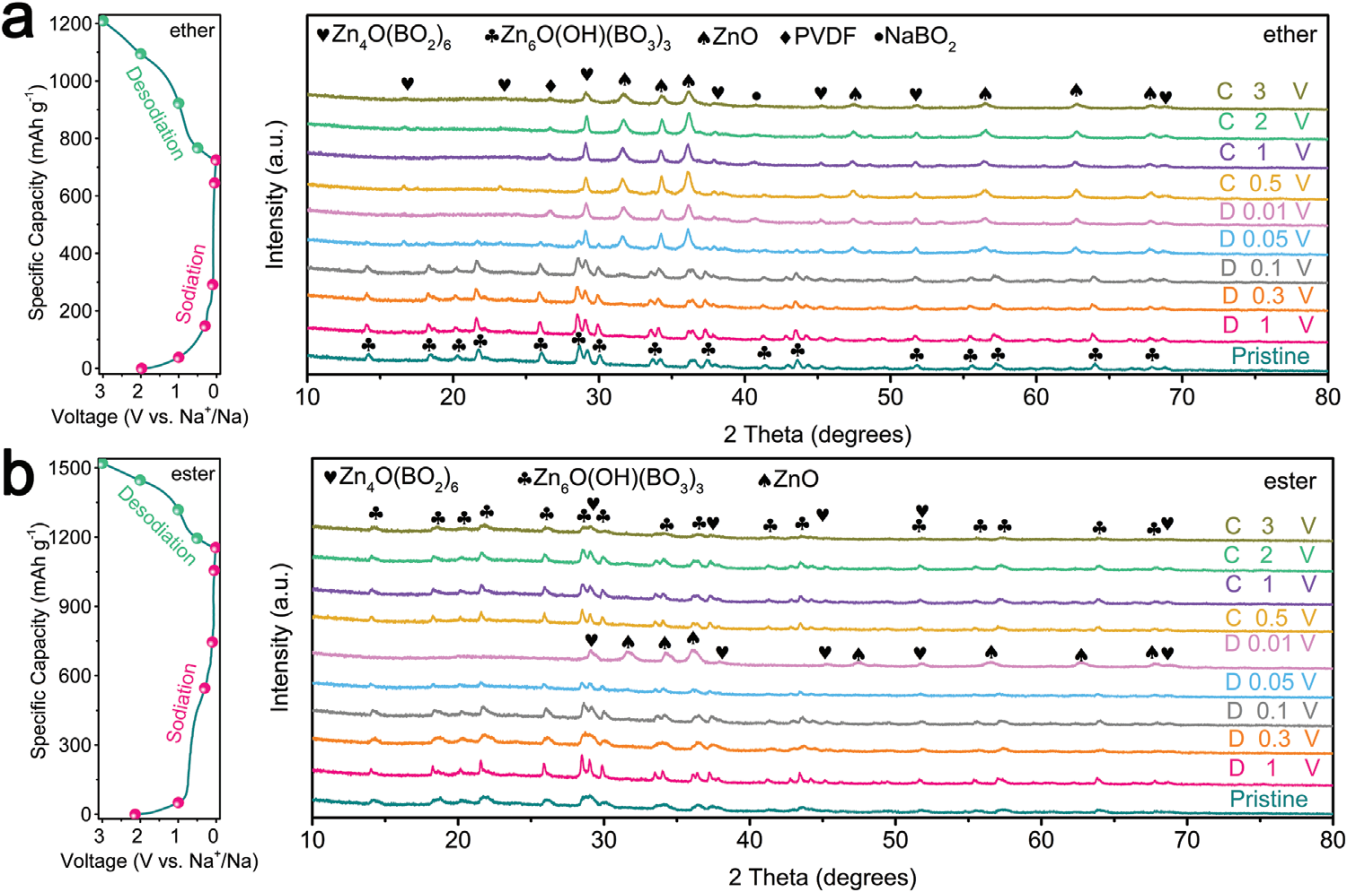
Na^+^ storage process analysis of CBZG as the half‐cell anode in ether‐ or ester‐based electrolyte. Discharge/charge profiles at 0.05 A g^−1^ and ex situ XRD patterns under various stages in a) ether‐based electrolyte and b) ester‐based electrolyte.

From the above results, we can find that CBZG anode possesses superior sodium storage performance in ether‐based electrolyte rather than ester‐based electrolyte, which can be attributed to the different sodium storage mechanisms and electrochemical kinetics processes between ether‐ and ester‐based electrolytes. Therefore, we employed ex situ XRD and XPS to study the electrode reaction process of CNZG anode in ether‐ and ester‐based electrolytes. **Figure** **5**a,b shows the ex situ XRD patterns of CNZG anode in ether‐ and ester‐based electrolytes at different discharge‐charge stages during the initial cycle of 0.05 A g^−1^. The pristine XRD patterns in ether‐ and ester‐based electrolytes are in accord with Figure 2a. As the voltage is discharged to 0.01 V in both electrolytes, the distinct diffraction peaks of Zn_6_O(OH)(BO_3_)_3_ phase undermine and finally disappear, ZnO, and amorphous‐like Zn_3_B_2_O_6_ are expected to appear, but the Zn_4_O(BO_2_)_6_ phase still exists, which suggests that Zn_6_O(OH)(BO_3_)_3_ is the active phase while Zn_4_O(BO_2_)_6_ is the inert phase during the conversion reaction. It is worth noting that the new phase of NaBO_2_ is appear in ether‐based electrolyte when discharged to 0.01 V. As the voltage is charged to 3 V, for ether‐based electrolyte, except for the presence of Zn_4_O(BO_2_)_6_, ZnO, and NaBO_2_, no new phase appears/disappears; for ester‐based electrolyte, the ZnO phase fades away, and the Zn_6_O(OH)(BO_3_)_3_ phase reappear.

In both electrolytes, when gradually discharged to 0.01 V, the ex situ XPS B 1s peak moves slowly to lower binding energy as the arrow trend in the Figures S11–S13 (Supporting Information), which is correlated to the BO_3_ with nonbridging oxygen and the reduction of B‐O bond caused by the bonding of Na^+^ with O^2−[^ ^15^ ^]^; when gradually charged to 3 V, the B 1s peak moves back to the original position. The pristine ex‐situ XPS Zn 2p peaks are at 1019.6 eV (Zn 2p_3/2_) and 1042.6 eV (Zn 2p_1/2_) in both electrolytes, implying the oxidation state of zinc in CBZG is +2 (Figures S11, S14, and S15, Supporting Information). After discharging to 0.01 V, the peaks of Zn 2p shift to 1018.5 eV (Zn 2p_3/2_) and 1041.6 eV (Zn 2p_1/2_) in ether‐based electrolyte, at 1019.2 eV (Zn 2p_3/2_) and 1042.2 eV (Zn 2p_1/2_) in ester‐based electrolyte, manifesting the reduction reaction from Zn^+2^ to Zn metal and the deeper conversion degree in ether‐based electrolyte, which may be ascribed to the faster de‐solvation rate. After charging to 3 V, the peaks of Zn 2p come to pristine state in both electrolytes, indicating the oxidation reaction of Zn metal and high reversibility. Interestingly, this reversible peak shift phenomenon is also found in N 1s and Na 1s in both electrolytes, as illustrated in Figure S16 (Supporting Information), implying a reversible process of conversion reaction, Na^+^ intercalation/deintercalation, or surface reaction with functional groups. As mentioned above, the possible conversion mechanisms of CBZG anode during discharge/charge progress in both electrolytes are as follows:

In ether‐based electrolyte:

(8) $$\begin{array}{ccc} {\mathrm{Zn}}_{6}O\left(\mathrm{OH}\right){\left({\mathrm{BO}}_{3}\right)}_{3}+{\mathrm{Na}}^{+}+e^{-} & \to & 3\mathrm{ZnO}+{\mathrm{Zn}}_{3}B_{2}O_{6}+{\mathrm{NaBO}}_{2}\\ & & +\;0.5H_{2}\left(\mathrm{irreversible}\right) \end{array}$$

(9) $${\mathrm{Zn}}_{3}B_{2}O_{6}+6{\mathrm{Na}}^{+}+6e^{-}↔3\mathrm{Zn}+3{\mathrm{Na}}_{2}O+B_{2}O_{3}\left(\mathrm{reversible}\right)$$

In ester‐based electrolyte:

(10) $$\begin{array}{ccc} {\mathrm{Zn}}_{6}O\left(\mathrm{OH}\right){\left({\mathrm{BO}}_{3}\right)}_{3}+{\mathrm{Na}}^{+}+e^{-} & ↔ & 3\mathrm{ZnO}+{\mathrm{Zn}}_{3}B_{2}O_{6}+{\mathrm{NaBO}}_{2}\\ & & +\;0.5H_{2}\left(\mathrm{reversible}\right) \end{array}$$

(11) $$\begin{array}{c} {\mathrm{Zn}}_{3}B_{2}O_{6}+6{\mathrm{Na}}^{+}+6e^{-}↔3\mathrm{Zn}+3{\mathrm{Na}}_{2}O+B_{2}O_{3}\left(\mathrm{reversible}\right) \end{array}$$

During discharging, the conversion process in ether‐based electrolyte includes two steps: 1) the ZnO, NaBO_2_, H_2_, and amorphous Zn_3_B_2_O_6_ are generated from Zn_6_O(OH)(BO_3_)_3_, 2) Zn_3_B_2_O_6_ turn into Zn, 3Na_2_O, B_2_O_3_, so does ester‐based electrolyte. During charging, in ether‐based electrolyte, the amorphous Zn_3_B_2_O_6_ reforms; in ester‐based electrolyte, the Zn_6_O(OH)(BO_3_)_3_ reforms. Therefore, during discharging, when the voltage gradually decreases, the sodium storage mechanism of CBZG anode is: 1) Na^+^ adsorption of open pores on the surface (3–1 V), 2) Na^+^ adsorption at defective graphitic layers (1–0.5 V), 3) Na^+^ intercalation between graphitic layers (0.5–0.01 V), 4) conversion reaction between Na^+^ and Zn_6_O(OH)(BO_3_)_3_ (0.3–0.01 V), and 5) pore filling with the formation of quasi metallic sodium (0.01 V, Figure S17, Supporting Information). Charging is the reverse process.

**Figure 6:**
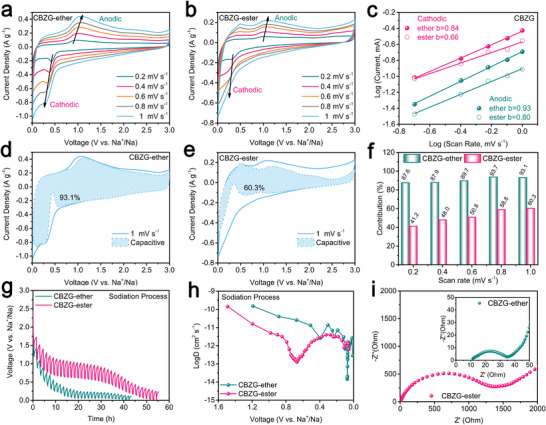
Kinetics analysis of Na^+^ storage of CBZG anode in ether‐ or ester‐based electrolyte. a, b) CV curves at different scan rates. c) *b*‐value calculation. d,e) Capacitive contributions at 1.0 mV s^−1^. f) Capacitive contributions at different scan rates. g) GITT potential profiles with a pulse current of 0.05 A g^−1^ for 0.5 h, followed by a 1.0 h relaxation process. h) Na^+^ diffusion coefficients calculated from the GITT potential profiles for the discharge process. i) Nyquist plots.

To gain further insight of the different sodium storage performance between ether‐ and ester‐based electrolytes, we adopted CV measurement to characterize the Na^+^ storage kinetics of CBZG anode in both electrolytes. In the region above 1.5 V, the CV curves show a rectangular‐like shape in both electrolytes, correlated to the reversible adsorption/desorption of Na^+^ in carbon pores and the pseudo‐capacitance via heteroatom functional groups (**Figure** **6**a,b). In the region below 1.5 V, the redox peaks relate to the Na^+^ sodiation/desodiation for zinc borate species and insertion/extraction or pore‐filling for carbon species. Based on the CV curves in Figure 6a,b, the Na^+^ storage kinetics progress is either capacitive‐controlled or diffusion‐controlled, which can be determined by formula *i = av^b^*, where *i* is current, *v* is scan rate, and *a, b* are adjustable constants.^[^ ^29^, ^36^ ^]^ The linear fitting of log (*i*) versus log (*v*) can quantify a *b*‐value—near 0.5 represents a diffusion‐controlled process (slow) while near 1.0 means a capacitive‐controlled process (fast). As displayed in Figure 6c, the cathodic and anodic *b*‐values in ether‐based electrolyte are 0.84 and 0.93, respectively, which are preferable than that of 0.66 (cathodic) and 0.80 (anodic) in ester‐based electrolyte, indicating an intrinsically fast kinetics process of CBZG anode in ether‐based electrolyte. To quantitatively calculate the capacitive/diffusion contribution of CBZG anode in both electrolytes, the Equation *i(V) = k_1_v+k_2_v^0.5^* is utilized, where *k_1_v* denotes the capacitive contribution and *k_2_v^0.5^* means diffusion contribution.^[^ ^33^, ^37^ ^]^ As shown in Figure 6d,e, the capacitive contribution at 1 mV s^−1^ of CBZG anode in ether‐based electrolyte has superior value of 93.1% (blue shadow), whereas that in ester‐based electrolyte encounters inferior value of 60.3% (blue shadow). Furthermore, the capacitive contribution in both electrolytes gradually increases with the enhancing of scan rate (Figure 6f). But the capacitive contribution value at each scan rate in ester‐based electrolyte is lower than that in ether‐based electrolyte, which is related to solvent molecule coordinating weakly with Na^+^ in ether‐based electrolyte, thus the carbon component adsorbs and the zinc borate reacts with sodium smoothly.

Figure 6g,h and Figure S18 (Supporting Information) exhibit galvanostatic intermittent titration technique (GITT) potential profiles with a pulse current of 0.05 A g^−1^ for 0.5 h (*τ*), followed by a 1.0 h relaxation process and Na^+^ diffusion coefficients calculated from Equation (12)^[^ ^29^, ^30^, ^37^, ^43^ ^]^:

(12) $$D_{{\mathrm{Na}}^{+}}=\frac{4}{\pi \tau }{\left(\frac{m_{B}V_{m}}{M_{B}S}\right)}^{2}{\left(\frac{\Delta E_{S}}{\Delta E_{\tau }}\right)}^{2},\tau \ll L^{2}/D_{{\mathrm{Na}}^{+}}$$

During sodiation process, CBZG anode shows a lower over‐potential and higher Na+ diffusion coefficients above 0.1 V and comparable performance below 0.1 V in ether‐based electrolyte, when compared with ester‐based electrolyte. During desodiation process, CBZG anode displays comparable Na^+^ diffusion coefficients in both electrolytes. Meanwhile, the electrochemical impedance spectroscopy (EIS) of CBZG anode has much lower R_ct_ (charge‐transfer resistance) of 21.9 Ω in ether‐based electrolyte than that of 1123.0 Ω in ester‐based electrolyte (Figure 6i and Table S4, Supporting Information). The straight slope of CBZG anode in ether‐based electrolyte is lower than that in ester‐based electrolyte (Figure S19, Supporting Information), indicating the higher Na^+^ diffusion in ether‐based electrolyte. The calculated Na^+^ diffusion coefficient (D Na^+^) for ether‐ and ester‐based electrolytes are 1.21×10^−12^ and 1.82×10^−15^ cm^2^ s^−1^, respectively. All the aforementioned kinetics test results indicate that the CBZG anodes show better performance in ether‐based electrolyte than ester‐based electrolyte, which can be ascribed to the reason as follows: 1) To some extent, Na^+^ storage kinetics is accelerated in ether‐based electrolyte by the fast formed thinner SEI layer that possesses more inorganic ingredients and dense morphology. 2) Na^+^ storage kinetics is promoted by the possibly quicker conversion reaction of zinc borate in ether‐based electrolyte. 3) Na^+^ storage kinetics is dominated by de‐solvation progress that is the key step of rate‐controlling, and ether‐based electrolyte has faster de‐solvation.

### 2.3 Structure and Electrochemical Characterizations of CBFG and CBCG

**Figure 7:**
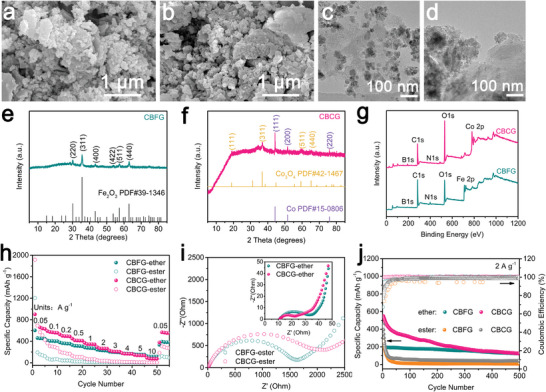
Structure characterizations and Na^+^ storage performance of CBFG and CBCG anodes in ether‐ or ester‐based electrolyte. a) SEM image of CBFG. b) SEM image of CBCG. c) TEM image of CBFG, d) TEM image of CBCG. e, f) XRD patterns. g) XPS survey spectra. h) Rate capability. i) Nyquist plots. j) Cycling performance at 2 A g^−1^.

According to the same experimental process as CBZG, we prepared CBFG and CBCG and tested their structures and sodium storage performance in both electrolytes. SEM and TEM were used to characterize the morphologies of CBFG and CBCG. As displayed in **Figure** **7**a–d, CBFG and CBCG show the analogous particle‐ and/or plate‐shaped morphologies in SEM images and exhibit the similar structure of disordered carbon embedding in oxide crystals in TEM images, which are created by the intercalator‐guided combustion activation without solid‐state synthesis (no corresponding borate was generated). This suggests that the structure similar to CBZG can only be formed by combining the abovementioned three synthesis conditions. XRD patterns identify the phases of CBFG and CBCG. Figure 7e displays one main phase of Fe_2_O_3_ (PDF#39‐1346) for CBFG and Figure 7f demonstrates two main phases of Co (PDF#15‐0806) and Co_3_O_4_ (PDF#42‐1467) for CBCG. The XPS survey spectra of CBFG and CBCG is showed in Figure 7g. Except for the difference of metal elements, both samples contain four elements: C, N, O, and B, and the corresponding elemental composition is in Table S1 (Supporting Information). Moreover, the elements are uniformly distributed on CBFG (C, N, O, B, and Fe) and CBCG (C, N, O, B, and Co), as shown in the elemental mapping images (Figure S20, Supporting Information). The above analyses manifest a N, B co‐doped carbon/Fe_2_O_3_ structure for CBFG and a N, B co‐doped carbon/Co/Co_3_O_4_ structure for CBCG. As shown in Raman spectra of Figure S21 (Supporting Information), the *I*_D_/*I*_G_ value of CBFG and CBCG is 2.08 and 2.71, respectively, indicating the introduction of B, N heteroatoms in carbon component create more defects,^[^ ^51^ ^]^ which is advantageous to boosting Na^+^ storage. The carbon contents of CBFG and CBCG are 19.7 wt.% and <10.3 wt.%, respectively, quantified by TG analysis under air flow (Figure S21, Supporting Information). The N_2_ adsorption–desorption isotherms and pore size distribution of CBFG and CBCG reveal that both samples have hierarchically porous structure that is caused by combustion activation (Figure S21, Supporting Information). Moreover, the BET specific surface area of CBFG and CBCG is 159 m^2^ g^−1^ and 202 m^2^ g^−1^, respectively, and the total pore volume of CBFG and CBCG is 0.151 and 0.325 cm^3^ g^−1^, respectively (Table S3, Supporting Information). As a result, anode materials with heteroatom‐doping and a high specific surface area can offer more defects for reversible surface adsorption, boost ion transport kinetics, and mitigate structural distortion, resulting in an enhanced rate capability and a structure stability of SIB.

The sodium storage performance of CBFG and CBCG anodes were also studied in ether‐ or ester‐based electrolyte. Ether‐based electrolyte has smaller first CV curve area at 0.2 mV s^−1^ than that of ester‐based electrolyte in both anodes, indicating a higher ICE, which may be attributed to the thinner SEI layer quickly formed in ether‐based electrolyte. Meanwhile, the almost overlapped 2nd and 3rd CV curves of CBFG and CBCG anodes in both electrolytes manifest the good electrochemical reversibility of both anodes (Figure S22, Supporting Information). What's more, the reversible redox peaks in the CV curves of both anodes in ether‐ or ester‐based electrolyte indicate the following conversion reactions:^[^ ^52^, ^53^ ^]^

(13) $${\mathrm{Fe}}_{2}O_{3}+6{\mathrm{Na}}^{+}+6e^{-}↔2\mathrm{Fe}+3{\mathrm{Na}}_{2}O$$

(14) $${\mathrm{Co}}_{3}O_{4}+8{\mathrm{Na}}^{+}+8e^{-}↔3\mathrm{Co}+4{\mathrm{Na}}_{2}O$$

As shown in Figure S23 (Supporting Information), the ICE of CBFG and CBCG anodes is 76.9%, 108.0% in ether‐based electrolyte and 8.7%, 29.2% in ester‐based electrolyte, respectively, calculated by the galvanostatic charge‐discharge profiles at 0.05 A g^−1^. The superior ICE in ether‐based electrolyte is consistent with CV result. Meanwhile, CBFG and CBCG anode have higher rate capability in ether‐based electrolyte than that in ester‐based electrolyte (Figure 7h; Figure S23 and Table S5, Supporting Information). The Na^+^ storage performance of CBFG and CBCG anodes is comparable to previous iron oxide or cobalt oxide based works in Table S6 (Supporting Information). As displayed in Figure 7i and Table S4 (Supporting Information), the much lower *R*_ct_ values of CBFG (20.0 Ω) and CBCG (12.1 Ω) anodes indicate their excellent kinetics in ether‐based electrolyte. The cycle test was also utilized to evaluate the performance of CBFG and CBCG anodes in ether‐ and ester‐based electrolytes (Figure 7j and Figure S24, Supporting Information). Both anodes in ether‐based electrolyte possess a preferable cycling performance at 2 A g^−1^ and 10 A g^−1^. Through the above analyses of the structure and performance in CBFG and CBCG anodes, we have proved the advantages of combustion activation in the construction of porous structure with heteroatoms again. Furthermore, the Na^+^ storage performance of ether‐based electrolyte is better than that of ester‐based electrolyte, which is in accord with the analysis results of CBZG anode.

### 2.4 Electrochemical Performance of CBZG, CBFG, and CBCG Based Full‐Cells

**Figure 8:**
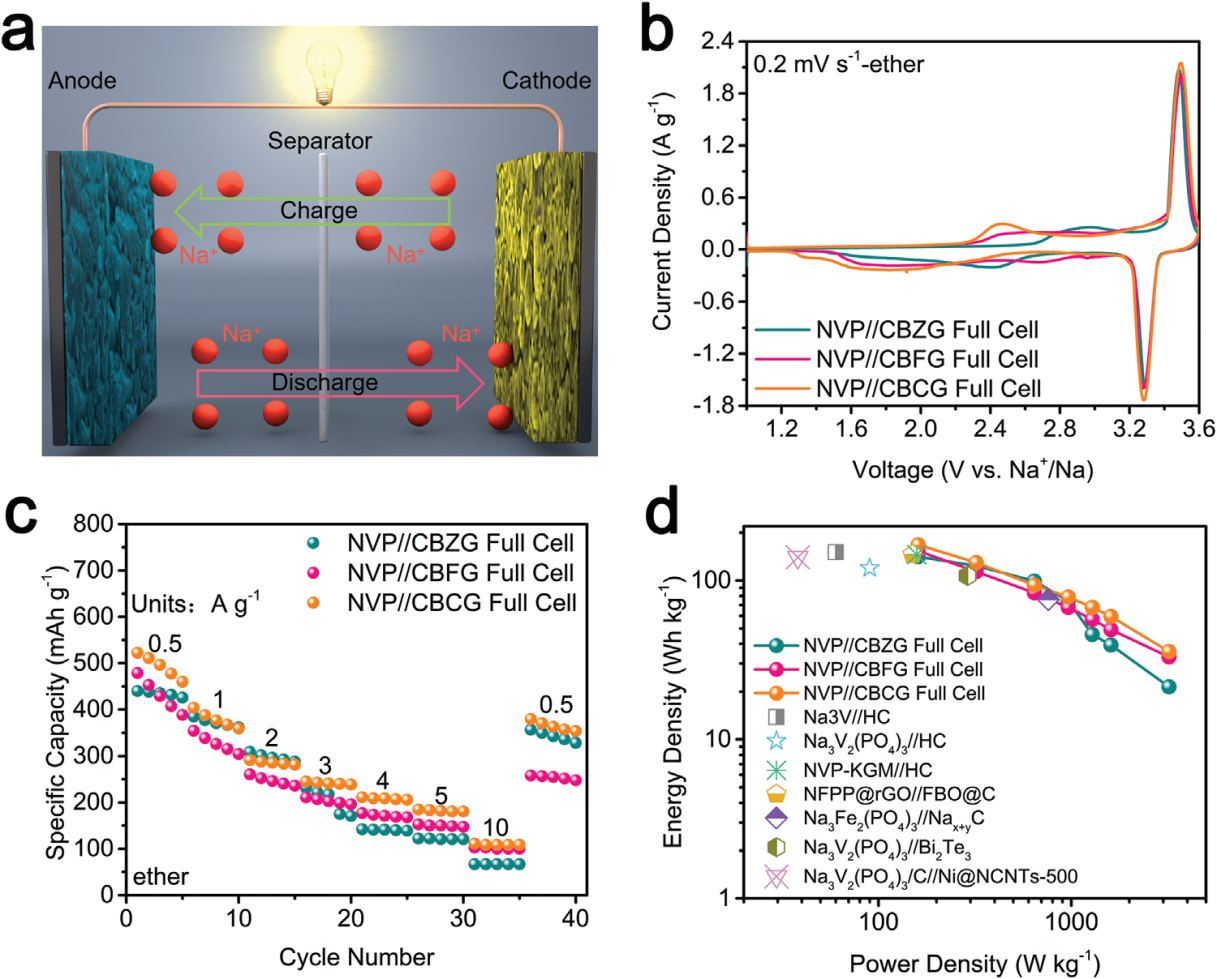
Electrochemical performance of NVP//CBZG, NVP//CBFG, and NVP//CBCG sodium‐ion full‐cells in ether‐based electrolyte; The current density and capacity of full cells are based on the anode mass only. a) Schematic illustration. b) CV curves at 0.2 mV s^−1^. c) Rate capability. d) Ragone plots comparison of different full‐cells.

Based on the better sodium storage performance of CBZG, CBFG, and CBCG anodes and Na_3_V_2_(PO_4_)_3_ (NVP) cathode in ether‐based electrolyte—the electrochemical analyses of NVP cathode are shown in Figure S25 (Supporting Information)—the NVP//CBZG, NVP//CBFG, and NVP//CBCG full‐cells were fabricated by using an ether‐based electrolyte, a cathode and a pre‐activated anode, which was cycled at the current of 0.05 A g^−1^ for three cycles in half‐cells before assembling. Meanwhile, the mass ratio of anode and cathode was set at 1:6 and the current density and capacity of full‐cells are calculated by the anode mass only. **Figure** **8**a shows the operation mechanisms of full‐cell: during charging, Na^+^ ions are extracted from NVP cathode first, and then pass‐through electrolyte, finally reach anode, while discharging is the reverse process. As exhibited in Figure 8b, two pairs of redox peaks on each CV curve of NVP//CBZG, NVP//CBFG, and NVP//CBCG full‐cells illustrate that both anode and cathode materials take part in the electrochemical reaction. Moreover, NVP//CBZG, NVP//CBFG, and NVP//CBCG full‐cells show superior rate capability in Figure 8c, Figure S26, and Table S7 (Supporting Information). Based on the total active mass of anode and cathode, the energy‐power densities of NVP//CBZG, NVP//CBFG, and NVP//CBCG full‐cells were calculated and showed in Figure 8d. The NVP//CBZG, NVP//CBFG, and NVP//CBCG full‐cells have energy densities of 141, 154, and 168 Wh kg^−1^ at power densities of 161 W kg^−1^, respectively; even the power densities reach 1607 W kg^−1^, the NVP//CBZG, NVP//CBFG, and NVP//CBCG full‐cells maintain energy densities of 39, 49, and 59 Wh kg^−1^, respectively, which is comparable among reported references.^[^ ^18^, ^54^, ^55^, ^56^, ^57^, ^58^, ^59^ ^]^ Moreover, the NVP//CBZG, NVP//CBFG, and NVP//CBCG full‐cells also display a stable lifespan—2000 cycles at 5 A g^−1^ (Figure S26, Supporting Information). In Figure S27 (Supporting Information), three fully charged full‐cells can drive LEDs (operating voltage: 3.0 V) with “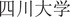,”“SCU,” and “SIB” logo well, suggesting that the NVP//CBZG, NVP//CBFG, and NVP//CBCG full‐cells can be applied in practical devices. Hence, both half‐ and full‐cells possess standout sodium storage properties in ether‐based electrolyte, confirming that the intercalator‐guided combustion activation is a very promising and universal synthesis method and ether‐based electrolyte is a potent kinetics accelerator for improving performance of sodium storage.

## 3 Conclusion

In summary, we developed a N, B co‐doped carbon/zinc borates (Zn_6_O(OH)(BO_3_)_3_ and Zn_4_O(BO_2_)_6_)—CBZG composite based on an intercalator‐guided combustion activation based solid‐state synthesis. The obtained CBZG anode shows high ICE (66.8%), reversible charge capacity (437.4 mAh g^−1^ at 0.05 A g^−1^), rate capability (193.4 mAh g^−1^ at 2 A g^−1^), and long‐term cycle life (capacity retention of 80.0% at 2 A g^−1^ over 500 cycles) in ether‐based electrolyte. The superior sodium storage properties in ether‐based electrolyte were also confirmed by counterpart anodes of N, B co‐doped carbon/Fe_2_O_3_ and N, B co‐doped carbon/Co/Co_3_O_4_. Taking CBZG as an example, we studied its sodium storage mechanism in ether‐ and ester‐based electrolyte by ex‐situ XRD and XPS; CV, GITT, and EIS. The possible reaction mechanism of CBZG anode during discharge/charge process is through Equations: Zn_6_O(OH)(BO_3_)_3_ + Na^+^ + e^−^ → 3ZnO + Zn_3_B_2_O_6_ + NaBO_2_ + 0.5H_2_, Zn_3_B_2_O_6_ + 6Na^+^ + 6e^−^ ↔ 3Zn + 3Na_2_O + B_2_O_3_ in ether‐based electrolyte; and Equations: Zn_6_O(OH)(BO_3_)_3_ + Na^+^ + e^−^ ↔ 3ZnO + Zn_3_B_2_O_6_ + NaBO_2_ + 0.5H_2_, Zn_3_B_2_O_6_ + 6Na^+^ + 6e^−^ ↔ 3Zn + 3Na_2_O + B_2_O_3_ in ester‐based electrolyte. Moreover, the electrochemical kinetic analysis suggests that CBZG anode has excellent Na^+^ storage kinetics in ether‐based electrolyte than ester‐based electrolyte. Therefore, our proposed synthesis method of borate composites exhibits their unique merits for drawing more attentions and inspiring more researches on exploring and synthesizing low‐cost and environment friendly borate materials with preferable sodium‐ion storage abilities for NIBs in the future.

## 4 Experimental Section

### Material Synthesis

N, B co‐doped carbon/zinc borate material was prepared by an intercalator‐guided combustion activation based solid‐state synthesis using hydroxypropyl cellulose (abbreviated as C) as a carbon source, boric acid (H_3_BO_3_, abbreviated as B) as intercalator and boron source, zinc nitrate hexahydrate (Zn(NO_3_)_2_⋅6H_2_O, abbreviated as Z) as oxidizer and Zn source, and glycine (abbreviated as G) as fuel and nitrogen source, along with the preparation of the carbon source‐free, the intercalator‐free, oxidizer‐free, or fuel‐free samples for comparison. Typically, 1 g hydroxypropyl cellulose, 5 g H_3_BO_3_, 12 mmol Zn(NO_3_)_2_⋅6H_2_O, and 24 mmol glycine were completely dissolved in 150 mL deionized water by stirring for 4 h in a water bath at room temperature, and followed by a water evaporation process at 90 °C under stirring. Then, the dry mixture was annealed in a tube furnace at 900 °C for 3 h with a heating rate of 5 °C min^−1^ under N_2_ atmosphere, and purified with sufficient hot water. After drying, the product was labeled as CBZG. For comparison, the CBZ sample was prepared without glycine, the BZ sample was prepared without hydroxypropyl cellulose and glycine, the CZG sample was prepared without H_3_BO_3_, the CZ sample was prepared without H_3_BO_3_ and glycine, the Z sample was prepared without hydroxypropyl cellulose, H_3_BO_3_ and glycine.With other conditions identical, the CBFG and CBCG samples were prepared by replacing Zn(NO_3_)_2_⋅6H_2_O with Fe(NO_3_)_3_⋅9H_2_O and Co(NO_3_)_2_⋅6H_2_O, respectively.

### Material Characterization

Thermogravimetric analysis (TG, TGA/DSC simultaneous thermal analyzer, TA Q600) was used to test the weight percent of carbon in the obtained composites in air flow and the pyrolysis process of precursor in N_2_ flow, from room temperature to 900 °C with a heating rate of 10 °C min^−1^. Scanning electron microscopy (SEM, Thermo Scientific Apreo 2C) and transmission electron microscopy (TEM, FEI Tecnai G2 F20) were used to characterize the morphology of the obtained composites. XRD (Rigaku Ultima IV with Cu‐K*α* radiation at 40 kV), Raman spectrometer (Horiba LabRAM HR Evolution, excitation wavelength: 532 nm), XPS (Thermo Scientific K‐Alpha), Fourier transform infrared spectroscopy (FTIR, Thermo Scientific Nicolet 6700), and automatic specific surface and porosity analyzer (Micromeritics ASAP 2460, N_2_ at 77K, Brunauer–Emmett–Teller (BET) method) were used to analyze the physical and chemical properties of the obtained composites. For ex situ XRD and XPS tests, the CBZG electrodes at various stages were cleaned in diethylene glycol dimethyl ether (DEGDME) or dimethyl carbonate (DMC) in an Ar‐filled glovebox, then encapsulated in a closed container before testing.

### Electrochemical Measurement

Half and full CR2032 coin cells were fabricated in an Ar‐filled glove box (O_2_ < 0.1 ppm, H_2_O < 0.1 ppm) to test Na^+^ storage performance of the as‐obtained composites. A slurry, including active material, acetylene black and poly(vinylidene fluoride) (PVDF) with mass ratio of 7:2:1 in *N*‐methyl‐2‐pyrrolidinone, was spread onto the stainless‐steel disc to prepare the working electrodes, and then dried at 100 °C in vacuum overnight. The active mass loading of each electrode is between 0.8 and 1.2 mg cm^−2^. For half‐cell, metallic sodium worked as counter electrode, 1 m NaFSI in ethylene carbonate and diethyl carbonate (EC: DEC = 1:1 in volume) or DEGDME served as electrolyte, and a glass fiber (Whatman, GF/D) functioned as separator. For full‐cell, the mass ratio of cathode (Na_3_V_2_(PO_4_)_3_) and anode (CBZG, CBFG, or CBCG) was set at 6:1, and electrolyte was 1 m NaFSI in DEGDME; The calculation of current density and capacity are based on the mass of CBZG only. Cyclic voltammetry (CV) and electrochemical impedance spectroscopy (EIS) were carried out on a CHI660E electrochemical workstation. Galvanostatic charge‐discharge tests (GCD) and galvanostatic intermittent titration technique (GITT) were performed on a NEWARE CT‐4008T battery measurement system. For GITT test, a series of current pulses at 0.05 A g^−1^ for 30 min followed by 60 min relaxation intervals was used. The voltage ranges of 0.01–3 V, 2–3.5/3.6 V, and 1–3.5/3.6 V were used for anode based half‐cells, cathode based half‐cells, and full‐cells, respectively.

## Conflict of Interest

The authors declare no conflict of interest.

## Supporting information

Supporting Information

## Data Availability

The data that support the findings of this study are available in the supplementary material of this article.
