# Supplementary material for: Combustion Activation Induced Solid‐State Synthesis for N, B Co‐Doped Carbon/Zinc Borate Anode with a Boosting of Sodium Storage Performance
Source: Adv Sci (Weinh). 2023 Mar 20;10(14):2207751. doi: 10.1002/advs.202207751 (PMC10190504; doi:10.1002/advs.202207751)
Supplement: Supplementary file 1 — Supporting Information [file ADVS-10-2207751-s001.pdf]

Supporting Information

Combustion activation induced solid-state synthesis for N, B co-doped carbon/zinc borate anode with a boosting of sodium storage performance

*Hao Zhang, Dingyue Zhang, Mingyi Guo, Zheng Huang, Xu Wang, Caiqin Gao, Fan Gao, Mauricio Terrones, and Yanqing Wang**

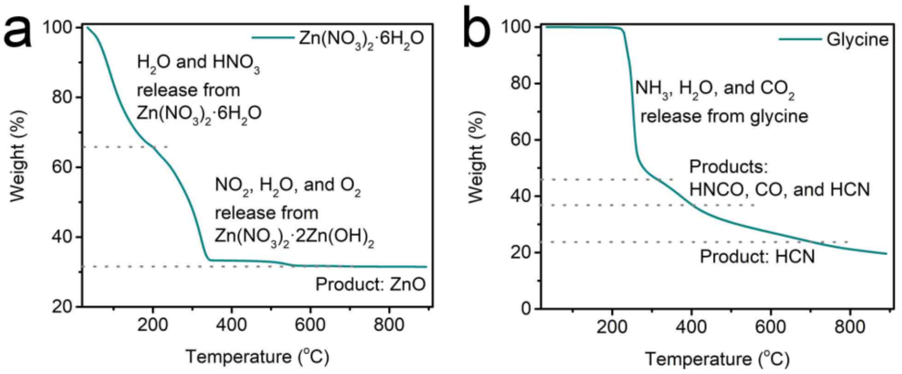

Figure S1. TG curves of a) $Zn(NO_3)_2{\cdot}6H_2O$ and b) glycine.

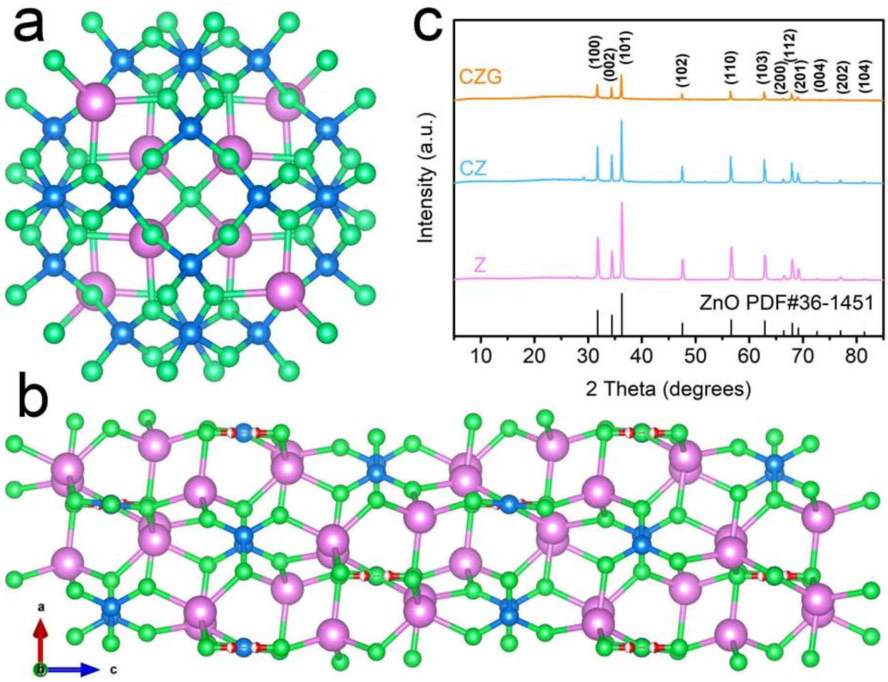

Figure S2. Crystal structure of a) $Zn_4O(BO_2)_6$ and b) $Zn_6O(OH)(BO_3)_3$ viewed along b-axis. c) XRD patterns of Z, CZ, and CZG.

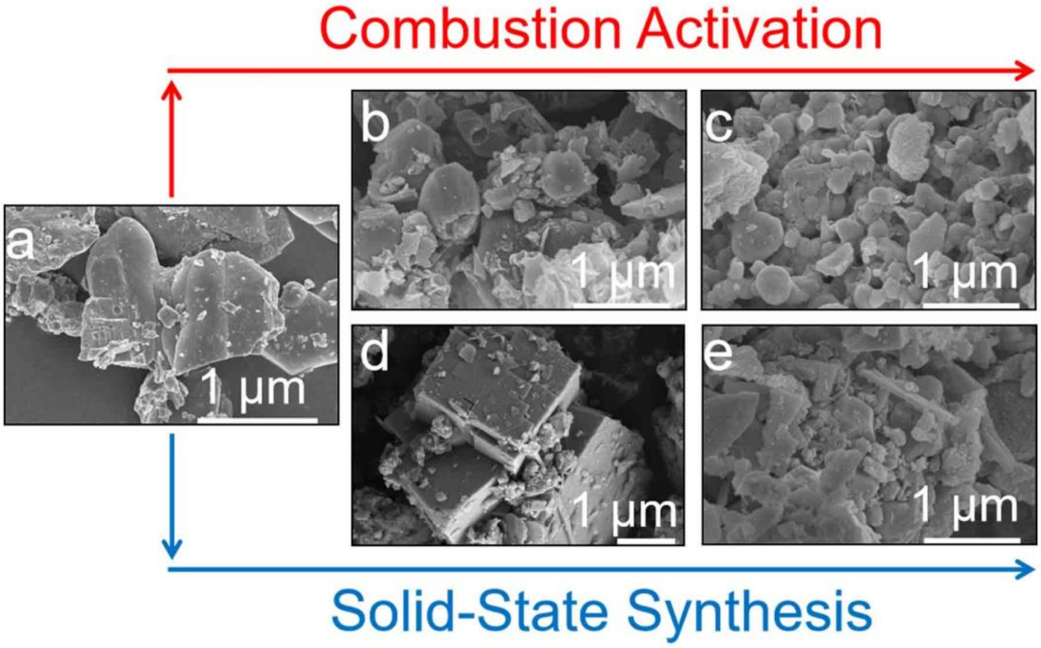

Figure S3. SEM images of a) Z, b) CZ, c) CZG, d) BZ, and e) CBZ.

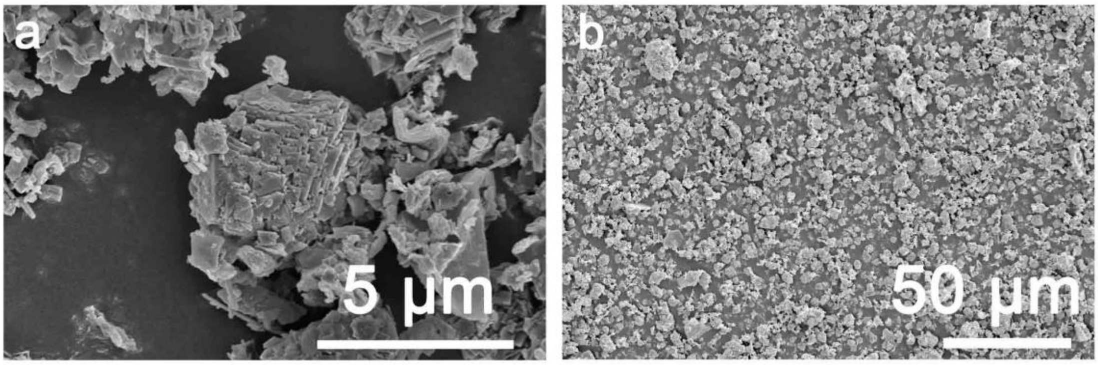

Figure S4. Lower magnification SEM images of CBZG.

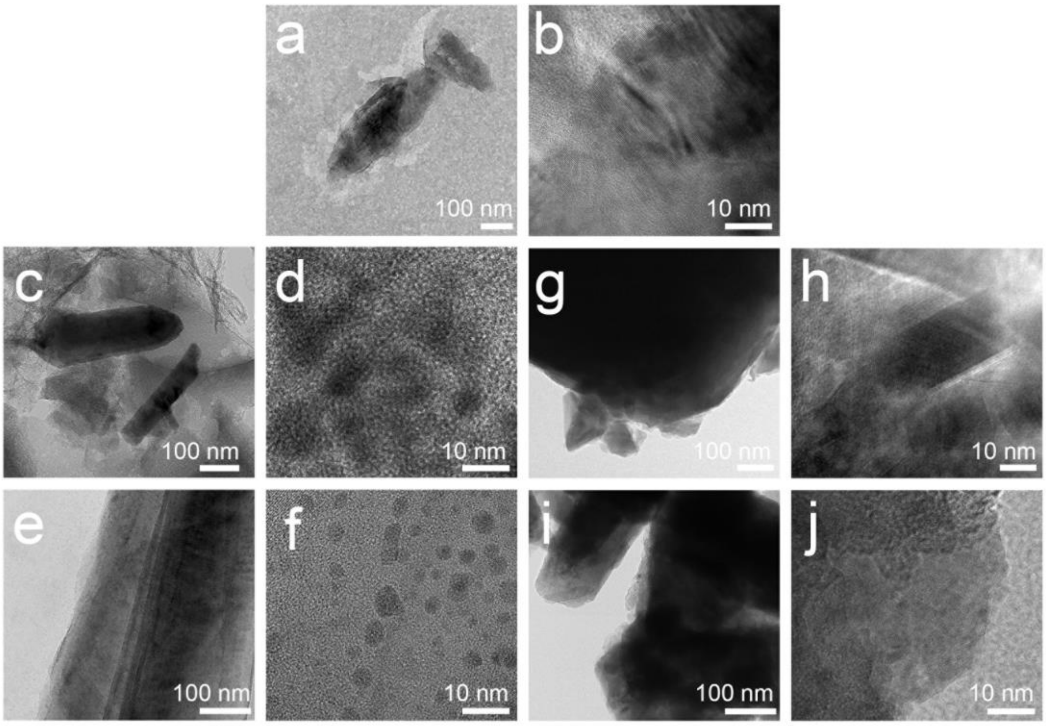

Figure S5. TEM and high-resolution TEM images of a, b) Z, c, d) CZ, e, f) CZG, g, h) BZ, and i, j) CBZ.

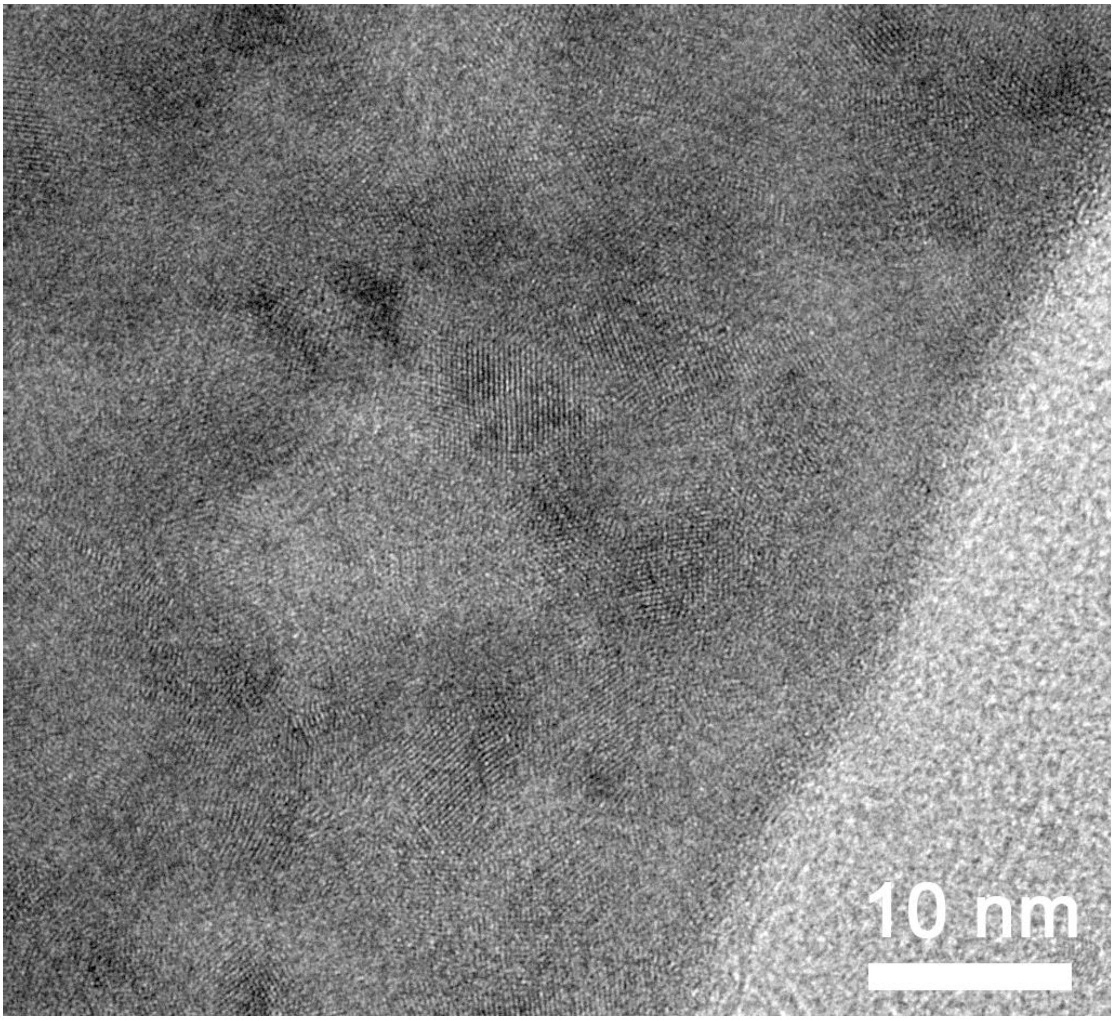

Figure S6. High-resolution TEM image of CBZG.

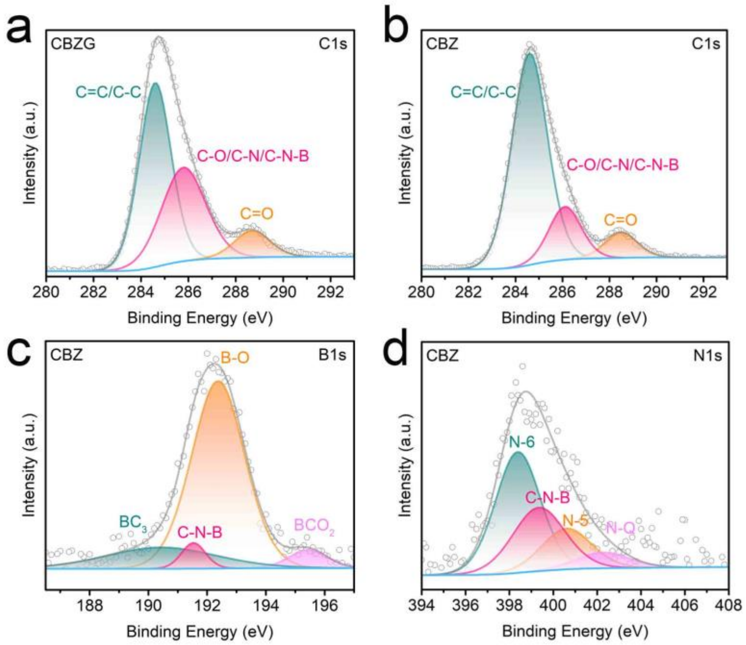

Figure S7. High-resolution C 1s XPS spectra of a) CBZG and b) CBZ. c, d) High-resolution B 1s and N 1s XPS spectra of CBZ.

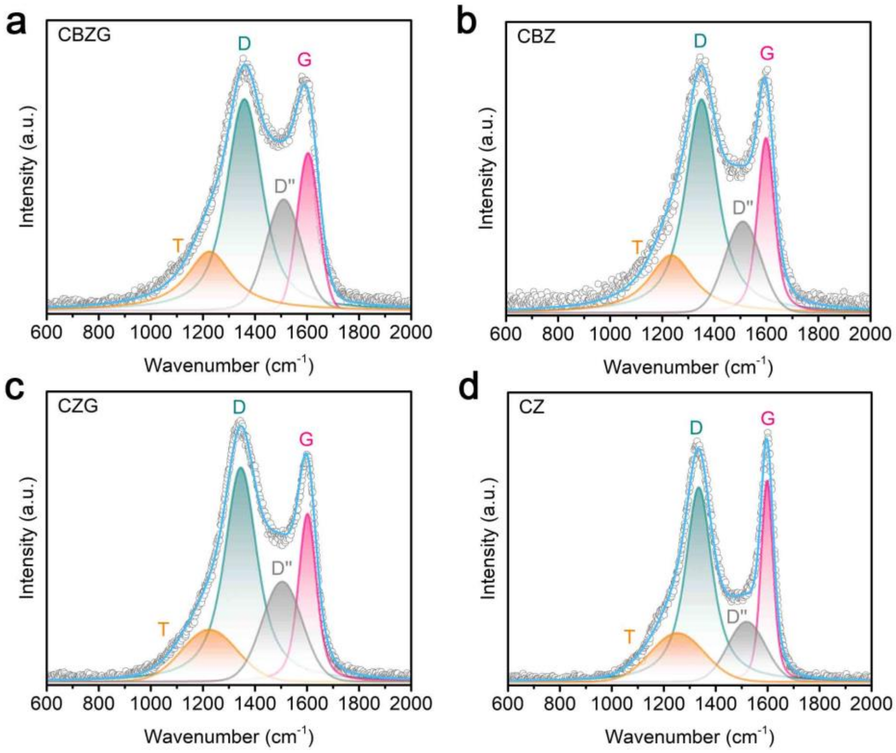

Figure S8. Fitted Raman spectra of a) CBZG, b) CBZ, c) CZG, d) CZ.

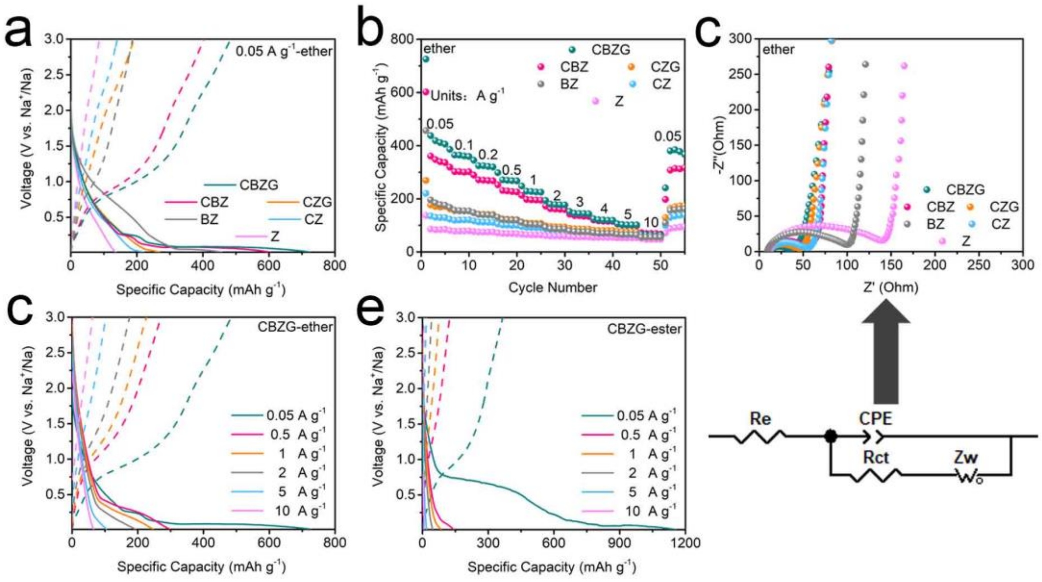

Figure S9. Na^+ storage performance of the obtained materials as the half-cell anode in ether- or ester-based electrolyte. a) Discharge/charge profiles at 0.05 A g^{-1}. b) Rate capability. c) Nyquist plots and equivalent circuit. d, e) Discharge/charge profiles at different current densities of CBZG.

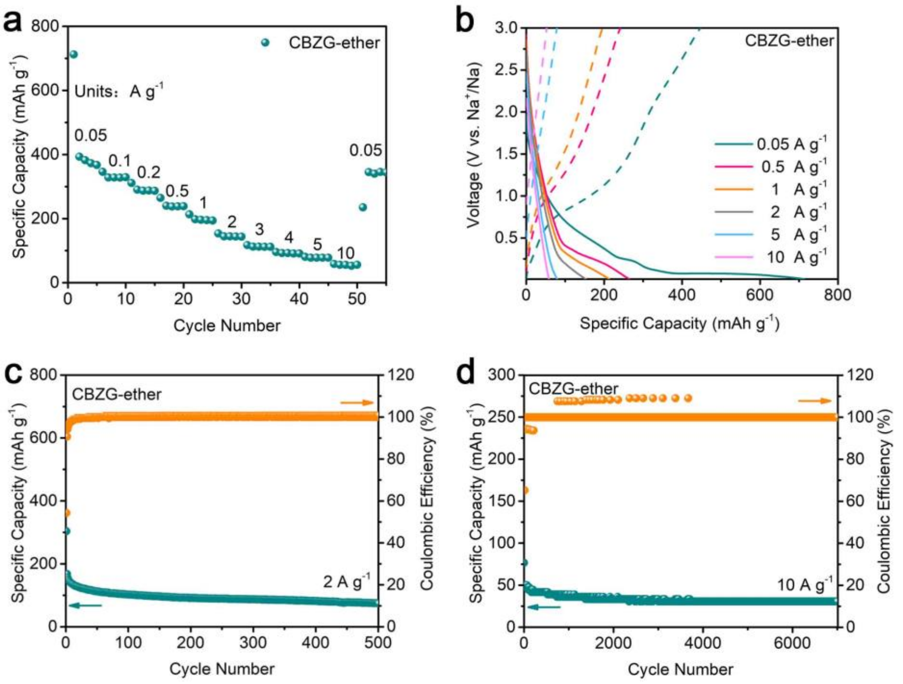

Figure S10. Na^+ storage performance of CBZG as the half-cell anode with a higher mass loading of ~2.0 mg cm^{-2} in ether-based electrolyte. a) Rate capability. b) Discharge/charge profiles at different current densities. Cycling performance at c) 2 A g^{-1} and d) 10 A g^{-1}.

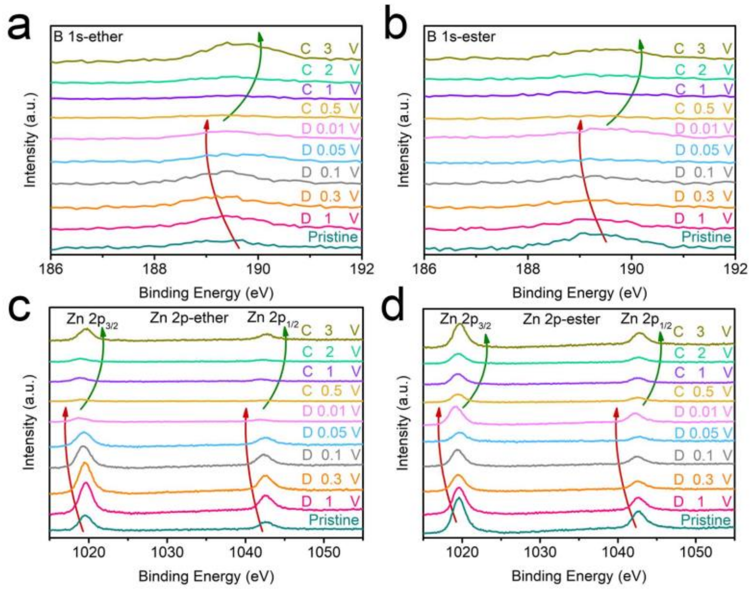

Figure S11. Na^+ storage process analysis of CBZG as the half-cell anode in ether- or ester-based electrolyte. Ex situ high-resolution XPS analysis at various stages for a, b) B 1s and c, d) Zn 2p.

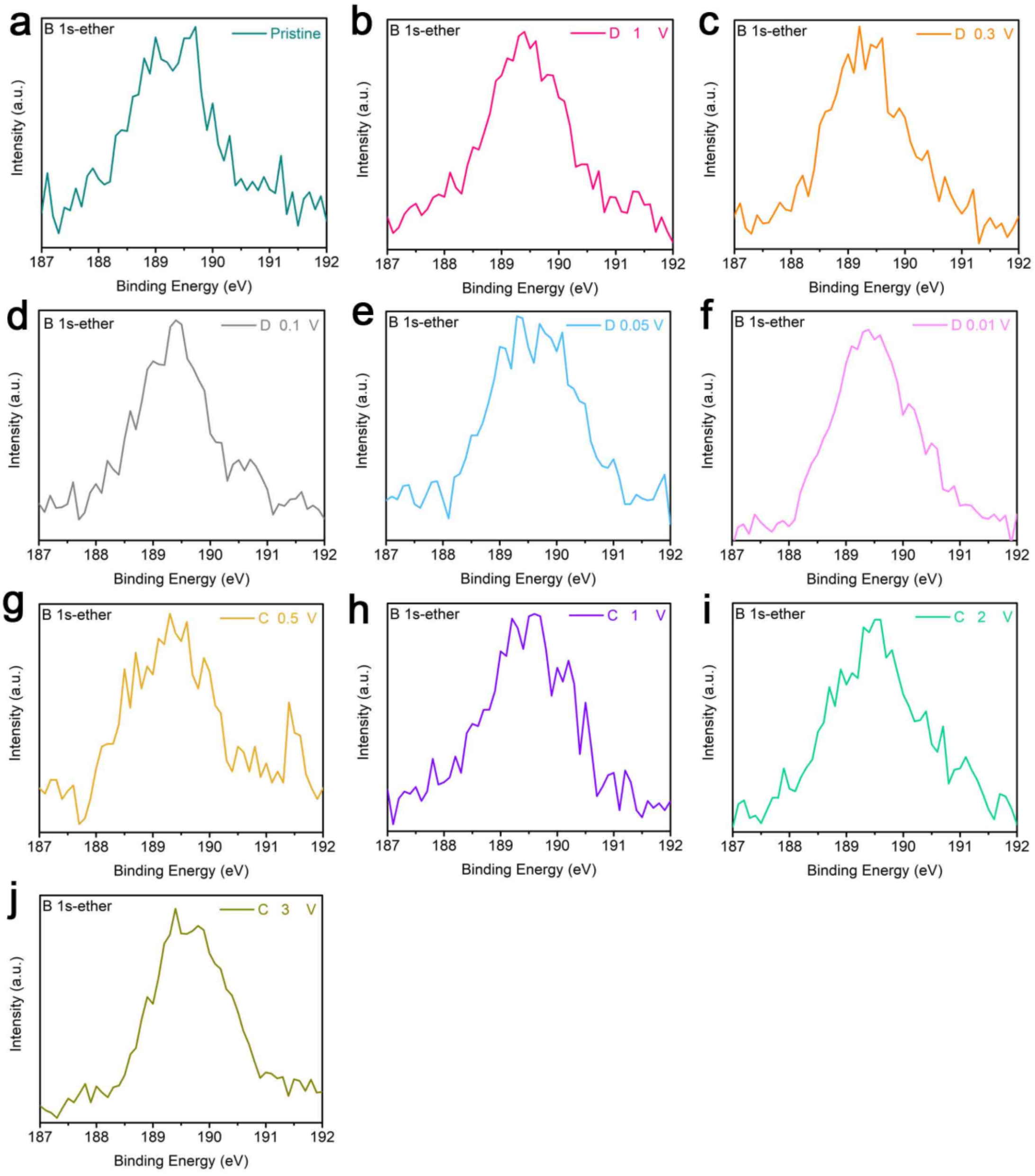

Figure S12. Ex situ high-resolution XPS analysis at various stages for B 1s in ether-based electrolyte.

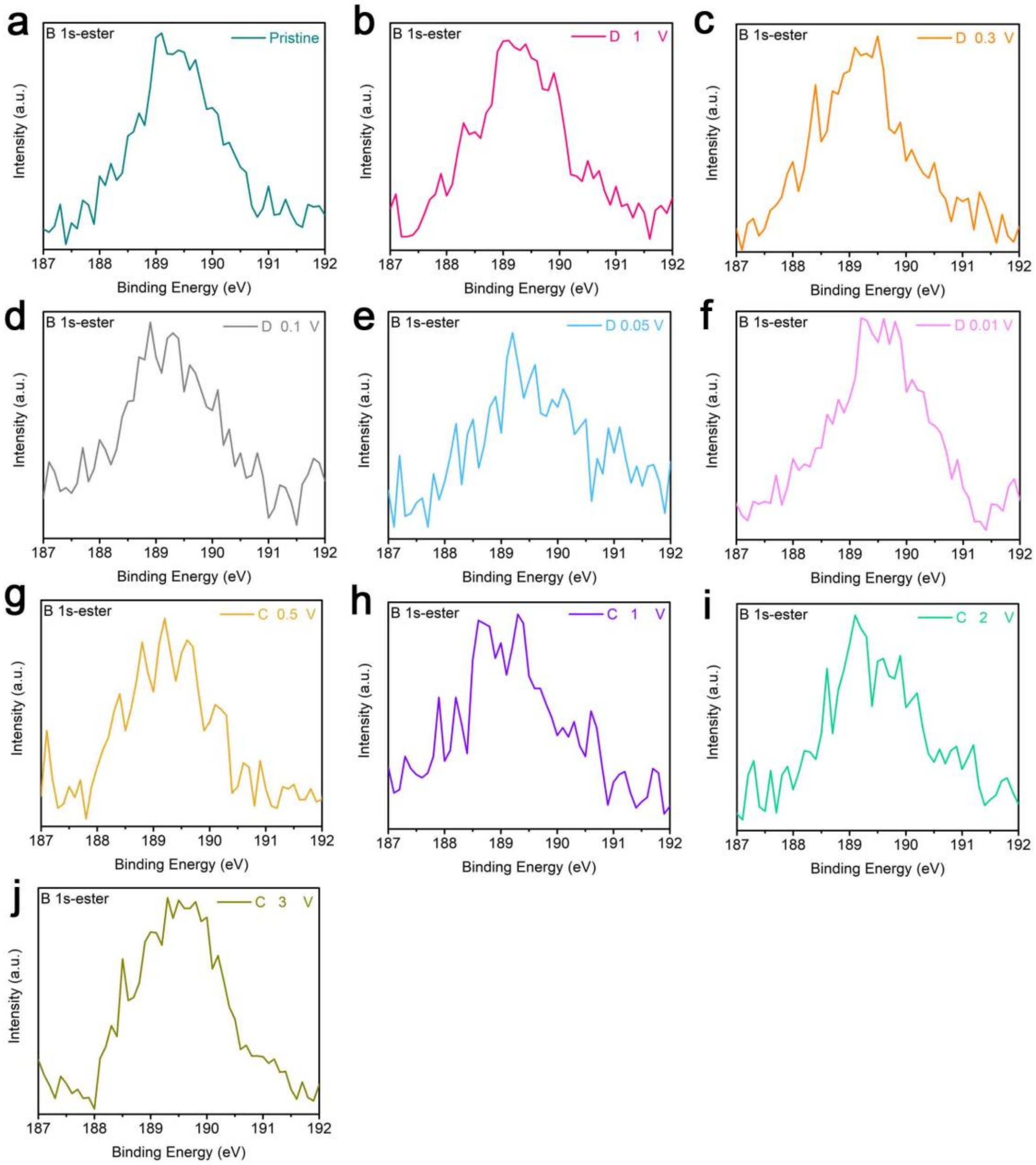

Figure S13. Ex situ high-resolution XPS analysis at various stages for B 1s in ester-based electrolyte.

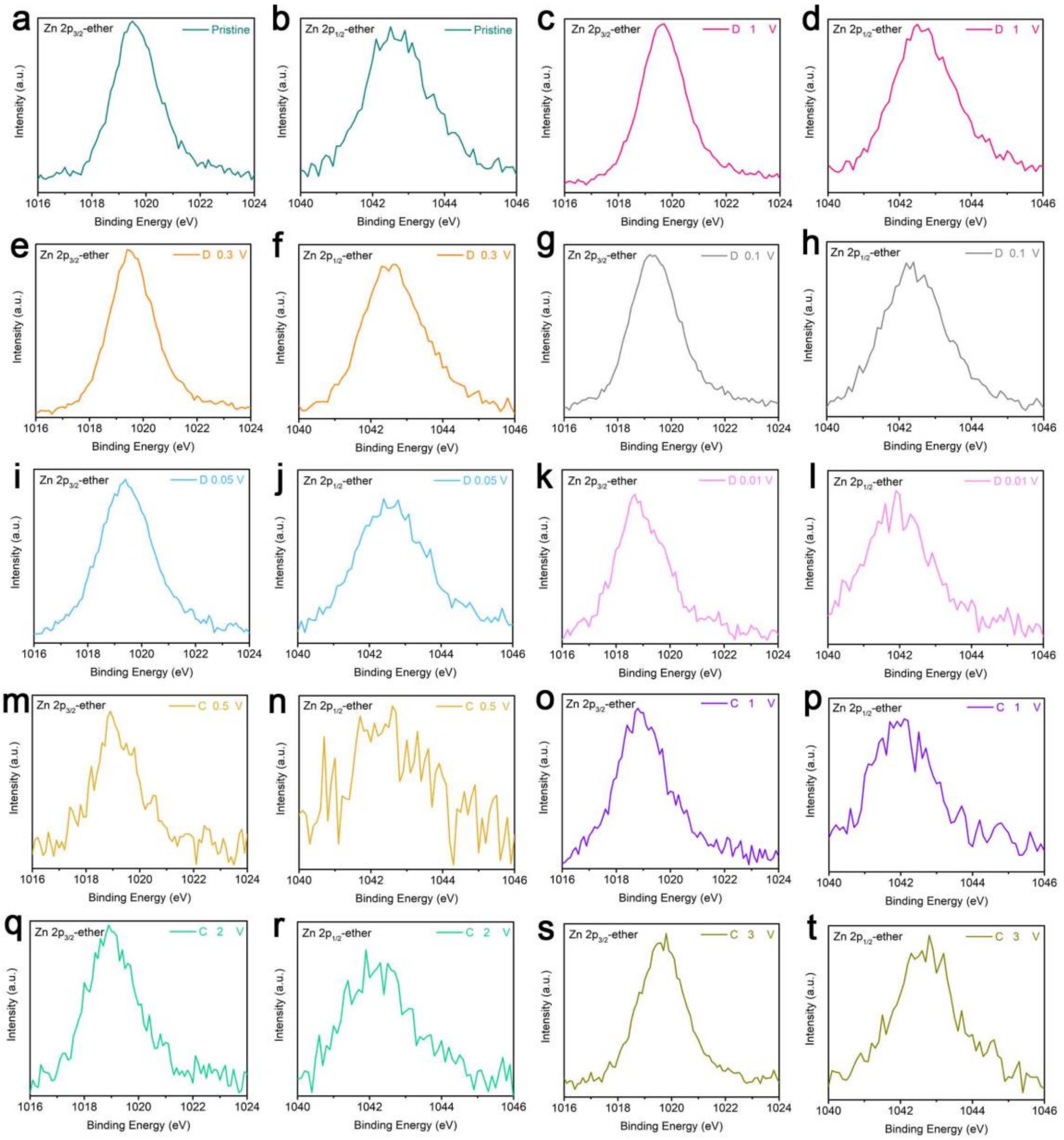

Figure S14. Ex situ high-resolution XPS analysis at various stages for Zn 2p in ether-based electrolyte.

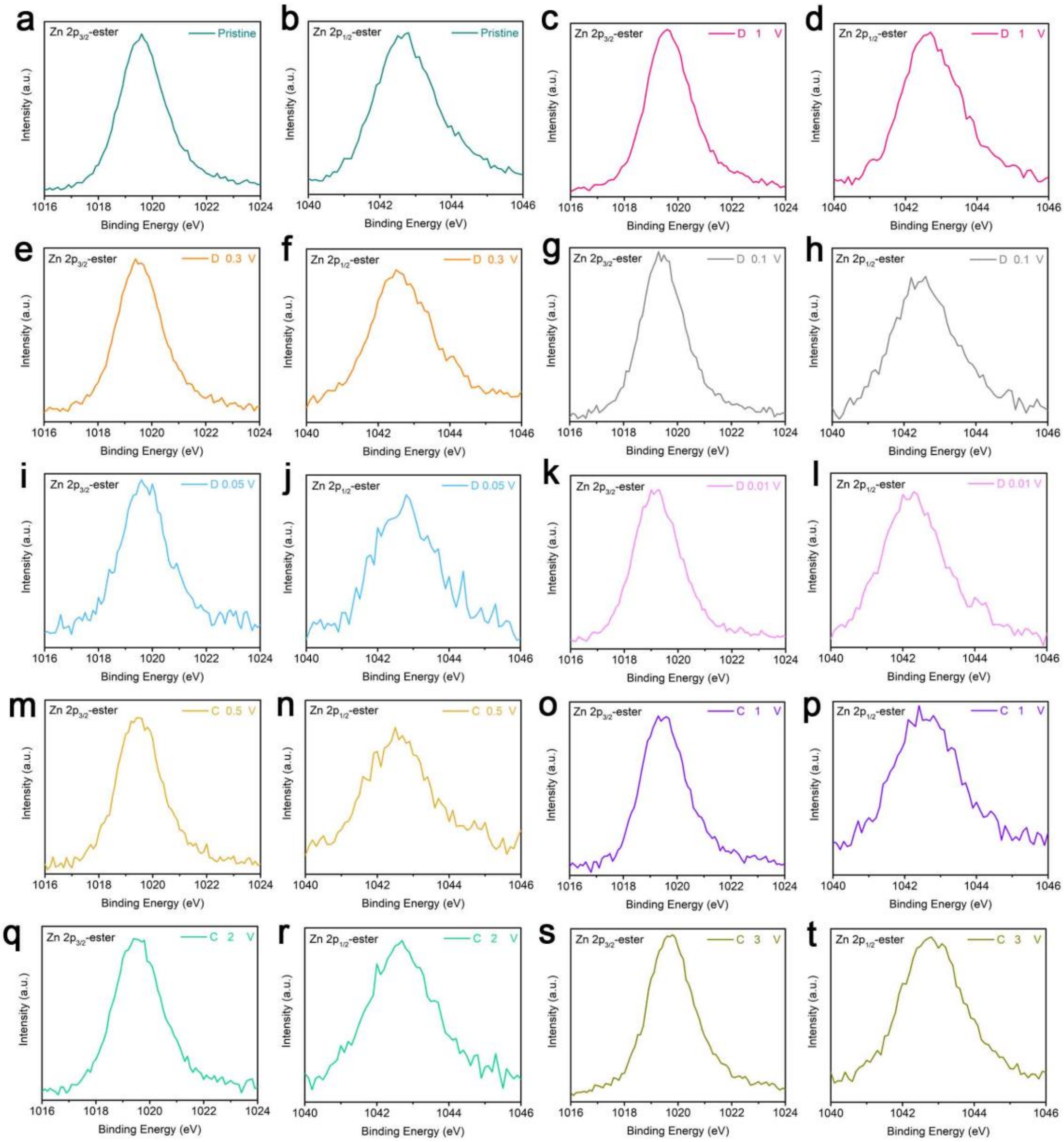

Figure S15. Ex situ high-resolution XPS analysis at various stages for Zn 2p in ester-based electrolyte.

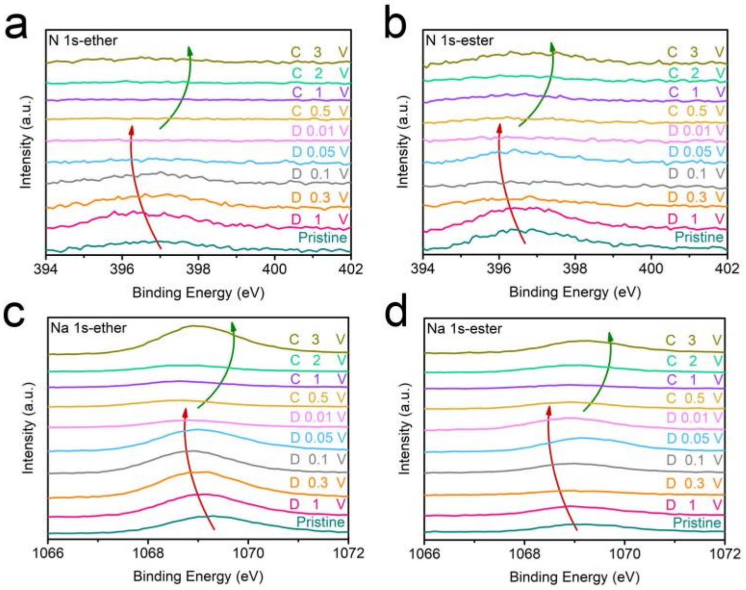

Figure S16. Na^+ storage process analysis of CBZG as the half-cell anode in ether- or ester-based electrolyte. Ex situ high-resolution XPS analysis at various stages for a, b) N 1s and c, d) Na 1s.

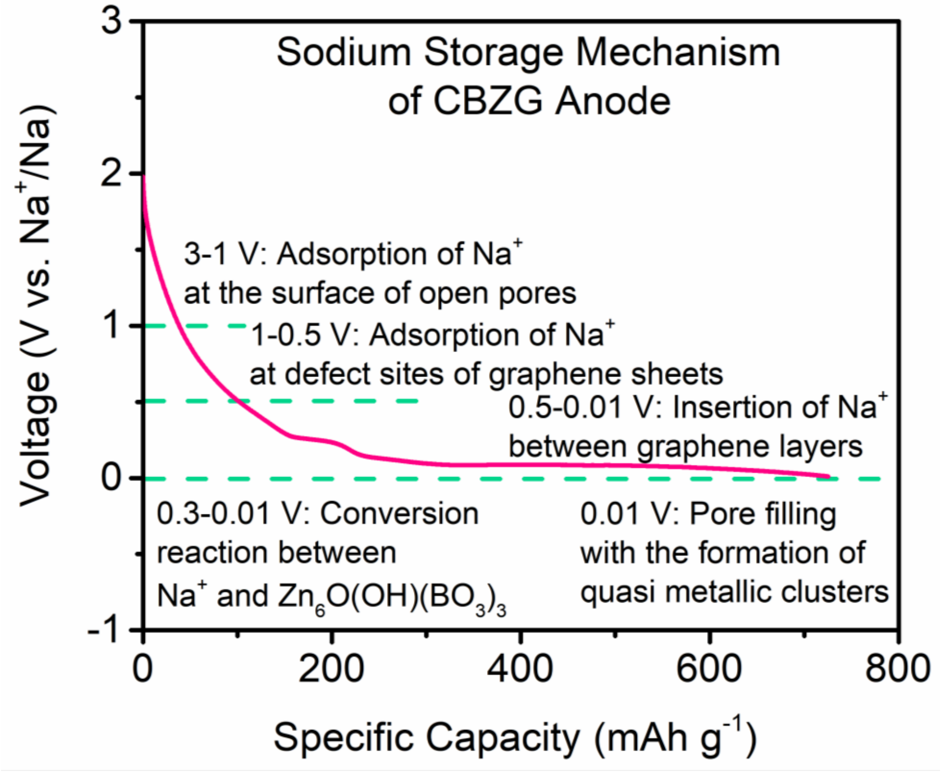

Figure S17. The sodium storage mechanism of CBZG anode.

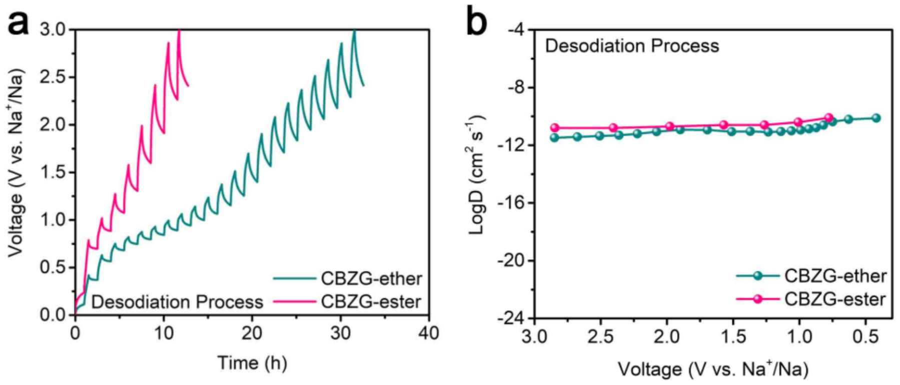

Figure S18. a) GITT potential profiles with a pulse current of 0.05 A g^{-1} for 0.5 h, followed by a 1.0 h relaxation process. b) Na^+ diffusion coefficients calculated from the GITT potential profiles for the charge process.

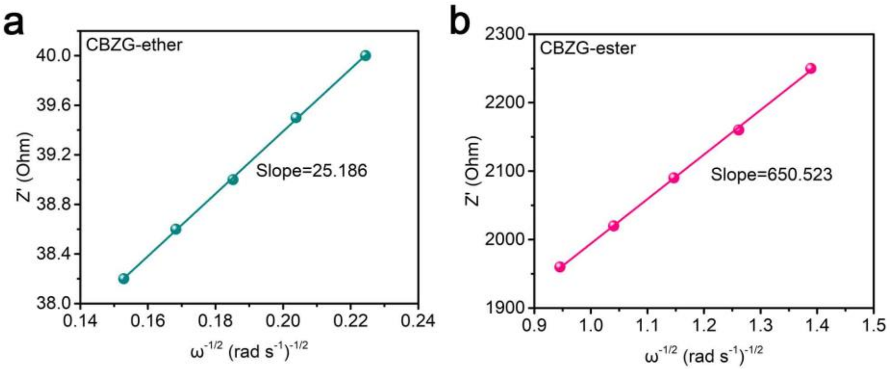

Figure S19. σ values (slope of the plot of Z' versus $\omega^{-1/2}$) of CBZG anode in a) ether-based electrolyte and b) ester-based electrolyte.

The diffusion coefficients of Na^+ ions were calculated from Electrochemical impedance spectra (EIS) according to the following equation [1]:

$$D_{Na^+} = \frac{R^2T^2}{2A^2n^4F^4C^2\sigma^2}$$

Where *R* is the gas constant, *T* is the Kelvin temperature, *A* is the electrode area, *n* is the number of electrons per molecule during the redox reaction, *F* is the Faraday constant, and *C* is the molar concentration of sodium ions. σ is the Warburg coefficient, which can be obtained from the slope of the plot of Z' versus $\omega^{-1/2}$ in **Figure S19**.

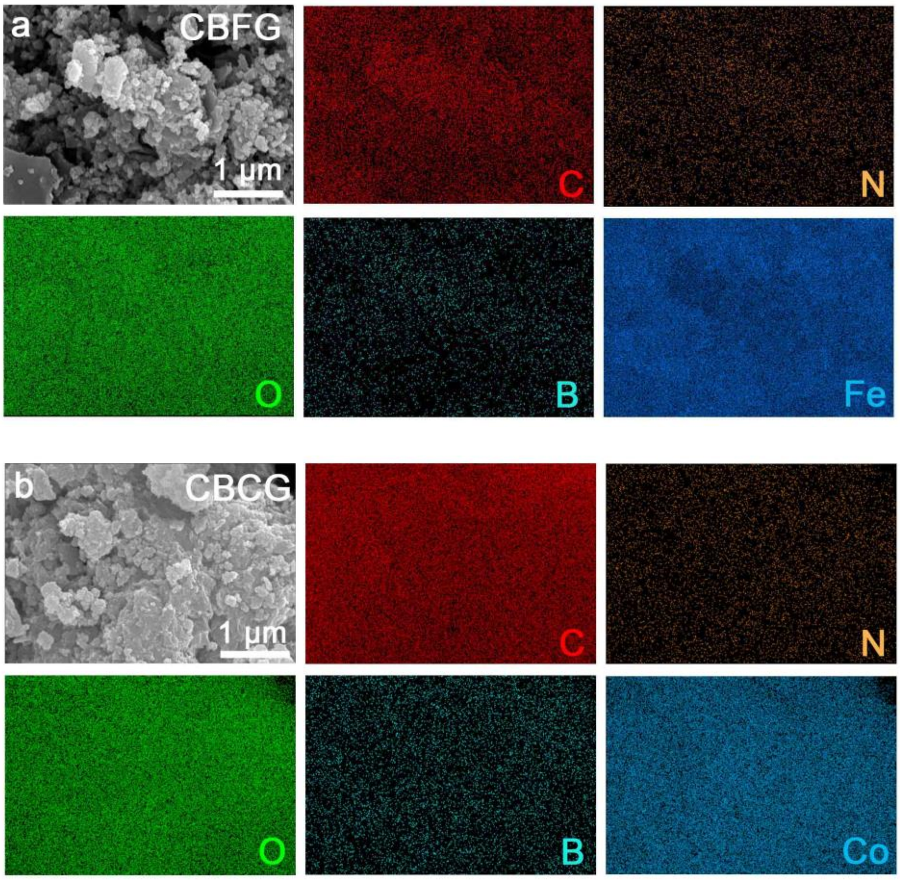

Figure S20. Element mapping images of a) CBFG and b) CBCG.

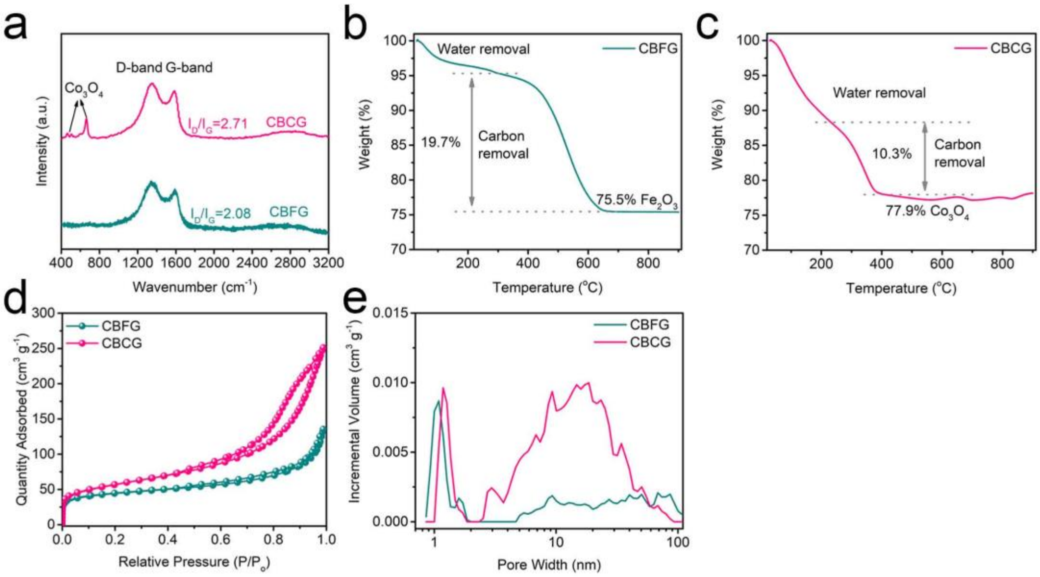

Figure S21. Structure characterizations of CBFG and CBCG. a) Raman spectra. TG curves of b) CBFG and c) CBCG. d) N_2 adsorption-desorption isotherms. e) Pore size distribution curves.

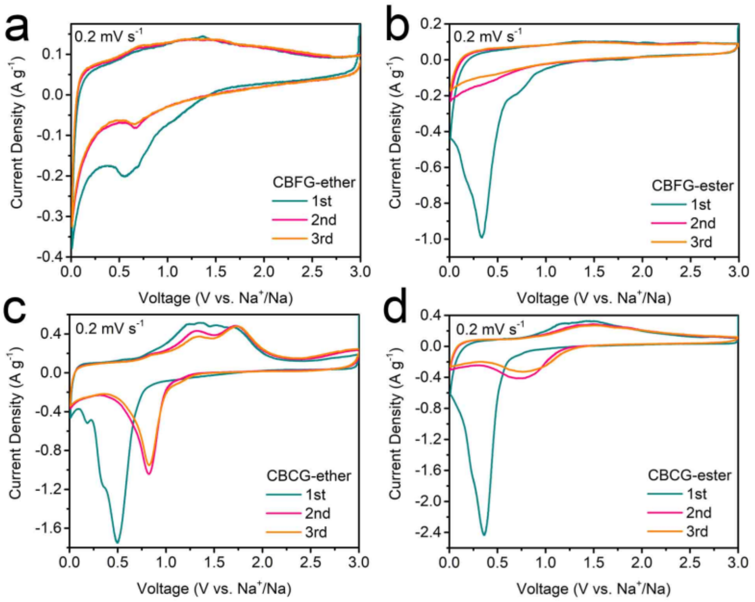

Figure S22. CV curves at 0.2 mV s^{-1} of CBFG and CBCG as the half-cell anode in ether- or ester-based electrolyte.

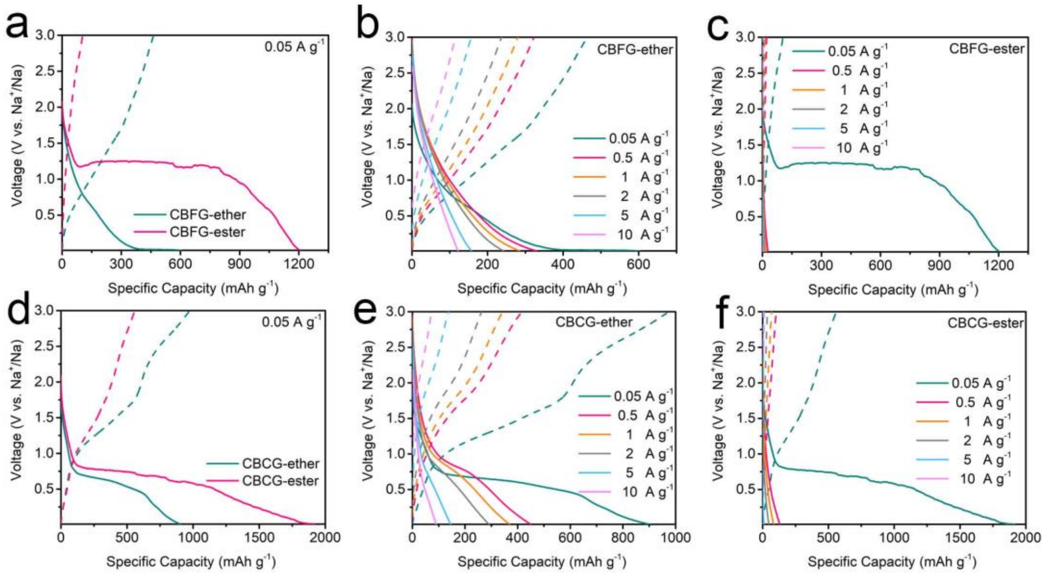

Figure S23. Discharge/charge profiles at different current densities of CBFG and CBCG as the half-cell anode in ether- or ester-based electrolyte.

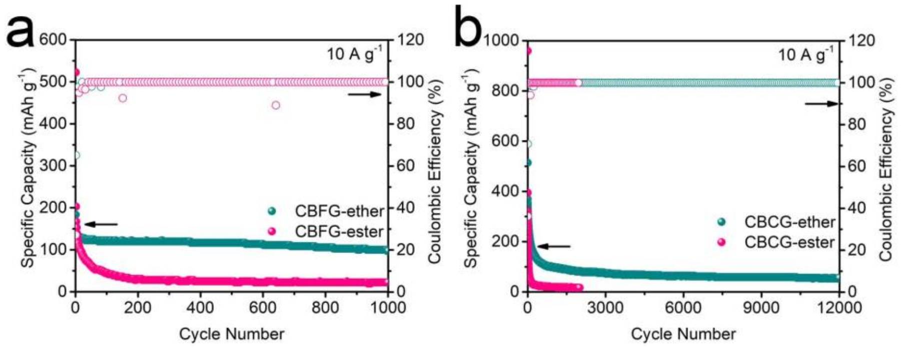

Figure S24. Cycling performance at 10 A g^{-1} of CBFG and CBCG as the half-cell anode in ether- or ester-based electrolyte.

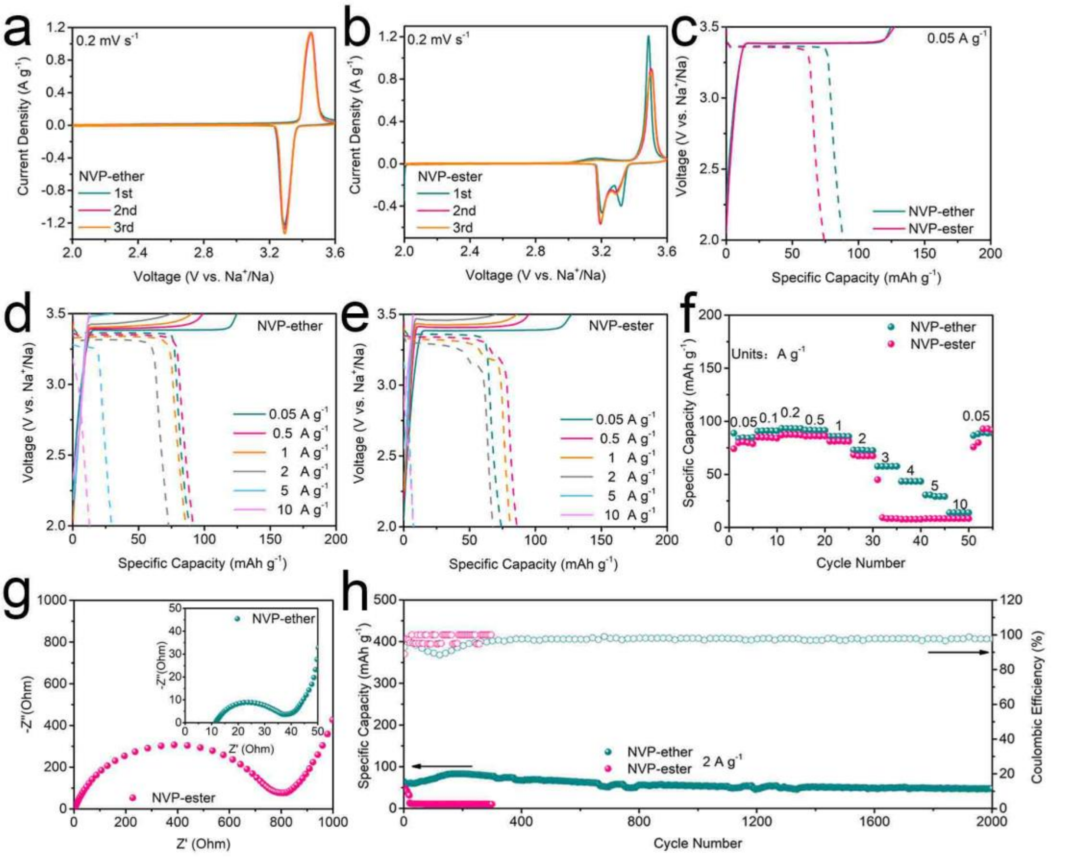

Figure S25. Na^+ storage performance of $Na_3V_2(PO_4)_3$ as the half-cell cathode in ether- or ester-based electrolyte. a, b) CV curves at 0.2 mV s^{-1}. c-e) Charge/discharge profiles at different current densities. f) Rate capability. g) Nyquist plots. h) Cycling performance at 2 A g^{-1}.

The sodium storage performance of commercial $Na_3V_2(PO_4)_3$ (NVP) cathode was also studied in ether- or ester-based electrolyte at 2-3.5/3.6 V. Ether-based electrolyte has higher ICE than ester-based electrolyte, confirmed by CV curves and the initial galvanostatic charge-discharge profiles at 0.05 A g^{-1}. In ether-based electrolyte, NVP cathode has rate capability of 88.8, 90.7, 93.3, 91.6, 85.9, 72.9, 57.5, 43.4, 30.6, 13.9, and 86.6 mAh g^{-1} at 0.05, 0.1, 0.2, 0.5, 1, 2, 3, 4, 5, 10, and 0.05 A g^{-1}; in ester-based electrolyte, NVP cathode has rate capability of 74.0, 85.0, 87.1, 86.0, 81.1, 68.3, 9.2, 7.8, 8.3, 8.3, and 75.8 mAh g^{-1} at the same current densities. NVP cathode has much lower R_{ct} (23.7 Ω) in ether-based electrolyte than that in ester-based electrolyte (734.4 Ω). Due to the intercalation property of NVP cathode, Na^+ need to de-solvate first, and

then intercalate into NVP cathode——the rate kinetics heavily hinge on the de-solvation rate.[2] Ether-based electrolyte shows a higher rate capability and lower R_{ct} than ester-based electrolyte, which also manifests the faster de-solvation in ether-based electrolyte. NVP cathode in ether-based electrolyte possess a superior cycling performance after 2 000 cycles at 2 A g^{-1} (84.7 % capacity retention), while in ester-based electrolyte has an inferior cycling performance below 300 cycles at 2 A g^{-1} (capacity close to zero). Through the above electrochemical analyses of NVP cathode, we have proved the advantages of ether-based electrolyte in sodium storage performance, which is coherent with the analysis results of anodes.

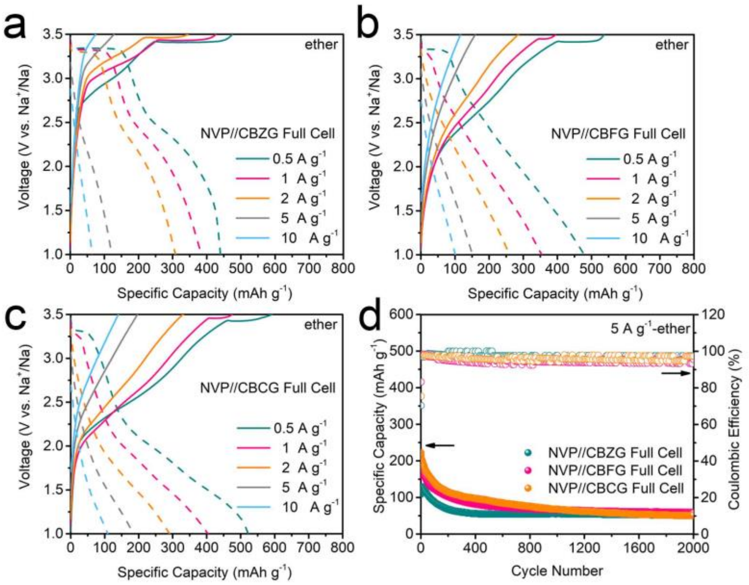

Figure S26. Charge/discharge profiles at different current densities of a) NVP//CBZG, b) NVP//CBFG, and c) NVP//CBCG sodium-ion full-cells in ether-based electrolyte. d) Cycling performance of sodium-ion full-cells at 5 A g^{-1} in ether-based electrolyte.

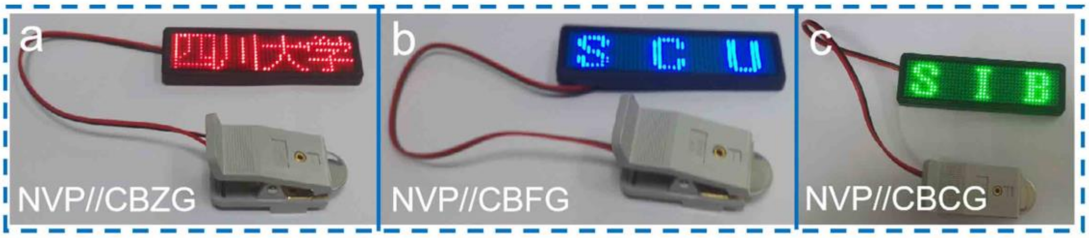

Figure S27. a-c) Photos show that full-cells can light up LED bulbs.

Table S1. The contents of C, N, O, B, Zn, Fe and Co in obtained various materials.

Samples	XPS composition (at%)						
	C	N	O	B	Zn	Fe	Co
CBZG	64.00	5.01	20.59	6.31	4.09	-	-
CBZ	61.37	1.87	24.93	6.07	5.76	-	-
BZ	-	-	44.00	18.22	13.31	-	-
CZG	77.77	2.33	16.54	-	3.36	-	-
CZ	79.29	-	17.06	-	3.65	-	-
CBFG	62.99	2.84	23.77	1.69	-	8.70	-
CBCG	52.17	0.47	33.64	4.88	-	-	8.85

Table S2. The fitting results of high-resolution XPS B 1s and N 1s for CBZG and CBZ.

Samples	B 1s (%)				N 1s (%)			
	BC_3	C-N-B	B-O	BCO_2	N-6	C-N-B	N-5	N-Q
CBZG	13.50	16.40	68.83	1.27	27.13	33.15	24.64	15.08
CBZ	17.25	4.56	73.08	5.11	46.77	28.59	17.49	7.15

Table S3. The specific surface area and pore volume of the obtained various materials.

Samples	S_{BET} (m^2 g^{-1})	V_t (cm^3 g^{-1})
CBZG	79	0.047
CBZ	36	0.028
BZ	0.4	0.002
CZG	238	0.116
CZ	70	0.055
Z	4	0.006
CBFG	159	0.151
CBCG	202	0.325

Table S4. R_e value and the R_{ct} value obtained from the equivalent circuit diagrams for various electrodes.

Electrolytes	Samples	R_e (Ω)	R_{ct} (Ω)
Ether	CBZG	11.4	21.9
	CBZ	10.7	39.3
	BZ	8.9	80.5
	CZG	11.1	31.3
	CZ	11.0	39.8
	Z	10.3	113.3
	CBFG	11.1	20.0
	CBCG	11.0	12.1
	NVP//CBZG	12.2	24.2
	NVP//CBFG	9.9	33.8
	NVP//CBCG	9.9	26.0
Ester	CBZG	4.5	1123.0
	CBFG	5.0	1636.0
	CBCG	4.3	2084.0

Table S5. The rate capability of CBFG and CBCG in ether- and ester-based electrolytes.

Samples	Capacity (mAh g^{-1})									
	0.05 A g^{-1}	0.1 A g^{-1}	0.2 A g^{-1}	0.5 A g^{-1}	1 A g^{-1}	2 A g^{-1}	3 A g^{-1}	4 A g^{-1}	5 A g^{-1}	10 A g^{-1}
CBFG-ether	460.5	415.2	377.9	329.9	285.1	243.0	205.1	177.9	158.4	122.3
CBCG-ether	690.6	589.7	527.4	449.5	370.1	291.1	225.9	178.9	145.8	91.7
CBFG-ester	193.4	81.6	52.1	28.2	17.8	9.5	5.8	4.4	4.2	4.2
CBCG-ester	690.6	364.6	205.1	130.2	83.9	47.3	27.5	17.8	12.5	5.6

Table S6. The capacity of C/Fe_2O_3 and C/Co/Co_3O_4 electrodes in comparison with previous iron oxide or cobalt oxide based works.

Sample	Capacity
**C/Fe_2O_3 (This work)**	460.5 mAh g^{-1} at 0.05 A g^{-1}<br>158.4 mAh g^{-1} at 5 A g^{-1}
GFe_2O_3-5[3]	445 mAh g^{-1} at 0.5 A g^{-1}<br>129 mAh g^{-1} at 5 A g^{-1}
Fe_2O_3:Ge NFs[4]	320 mAh g^{-1} at 0.05 A g^{-1}<br>140 mAh g^{-1} at 2 A g^{-1}
α- Fe_2O_3:Mn@rGO[5]	56 mAh g^{-1} at 1 A g^{-1}
4Fe_3O_4-CNFs[6]	431.1 mAh g^{-1} at 0.1 A g^{-1}

Table S7. The rate capability of NVP//CBZG, NVP//CBFG, and NVP//CBCG full-cells in ether-based

α-Fe_2O_3/γ-Fe_2O_3/Fe/C[7]	301 mAh g^{-1} at 0.1 A g^{-1}
Fe_2O_3@C[8]	434.4 mAh g^{-1} at 0.2 A g^{-1}
Ag@Fe_3O_4[9]	278 mAh g^{-1} at 0.2 C
Fe_2O_3-AHP[10]	354.3 mAh g^{-1} at 0.1 A g^{-1}
Fe_3O_4@C/rGO[11]	366 mAh g^{-1} at 0.1 A g^{-1}<br>125 mAh g^{-1} at 5 A g^{-1}
Fe_2O_3/NGC[12]	404.6 mAh g^{-1} at 0.25 A g^{-1}
**C/Co/Co_3O_4 (This work)**	690.6 mAh g^{-1} at 0.05 A g^{-1}<br>291.1 mAh g^{-1} at 2 A g^{-1}
Co_3O_4/N-CNS[13]	533 mAh g^{-1} at 0.05 A g^{-1}<br>191 mAh g^{-1} at 1 A g^{-1}
3D-CoO-NrGO[14]	445 mAh g^{-1} at 0.025 A g^{-1}<br>135 mAh g^{-1} at 5 A g^{-1}
Cu-Co_3O_4@Void@NC[15]	501 mAh g^{-1} at 0.2 A g^{-1}<br>301 mAh g^{-1} at 2 A g^{-1}
Co_3O_4@N-CNFs[16]	520 mAh g^{-1} at 0.1 A g^{-1}<br>205 mAh g^{-1} at 3.2 A g^{-1}
$ZnCo_2O_4$/RGO[17]	447.53 mAh g^{-1} at 0.1 A g^{-1}<br>214.73 mAh g^{-1} at 1.6 A g^{-1}
$NiCo_2O_4$/CTBs[18]	473 mAh g^{-1} at 0.1 A g^{-1}<br>236 mAh g^{-1} at 5 A g^{-1}
$Zn_xCo_{3-x}O_4$[19]	345 mAh g^{-1} at 0.2 A g^{-1}<br>142 mAh g^{-1} at 2 A g^{-1}
$CoMoO_4$[20]	231.3 mAh g^{-1} at 0.2 A g^{-1}<br>131.6 mAh g^{-1} at 2 A g^{-1}

$Fe_{0.8}CoMnO_4$[21]	396 mAh g^{-1} at 0.2 A g^{-1}<br>234 mAh g^{-1} at 2 A g^{-1}
Co_3O_4/ZnO[22]	242 mAh g^{-1} at 2 A g^{-1}

electrolytes.

Samples	Capacity (mAh g^{-1})						
	0.5 A g^{-1}	1 A g^{-1}	2 A g^{-1}	3 A g^{-1}	4 A g^{-1}	5 A g^{-1}	10 A g^{-1}
NVP//CBZG	440.1	385.2	309.1	231.8	142.3	122.3	66.7
NVP//CBFG	478.7	354.3	260.6	211.0	176.7	152.8	102.8
NVP//CBCG	522.5	403.9	291.4	245.2	211.3	184.8	111.2

References

[1] S. Huang, D. Yang, X. Qiu, W. Zhang, Y. Qin, C. Wang, C. Yi, Adv. Funct. Mater. 2022, *32*, 2203279.

[2] X. Yi, X. Li, J. Zhong, S. Wang, Z. Wang, H. Guo, J. Wang, G. Yan, *Adv. Funct. Mater.* **2022**, *32*, 2209523.

[3] Y. Zhang, Q. Wang, K. Zhu, K. Ye, G. Wang, D. Cao, J. Yan, *Chem. Eng. J.* **2022**, *428*, 131204.

[4] B. Petrovičovà, C. Ferrara, G. Brugnetti, C. Ritter, M. Fracchia, P. Ghigna, S. Pollastri, C. Triolo, L. Spadaro, R. Ruffo, S. Santangelo, *Appl. Sci.* **2021**, *11*, 1483.

[5] V. Modafferi, C. Triolo, M. Fiore, A. Palella, L. Spadaro, N. Pianta, R. Ruffo, S. Patane, S. Santangelo, M. G. Musolino, *Nanomaterials (Basel)* **2020**, *10*, 1588.

[6] Y. Huang, J. Zhou, Y. Zhang, L. Yan, S. Bao, Y. Yin, J. Lu, *J. Alloys Compd.* **2022**, *918*, 165672.

[7] L. Yin, Y. Pan, M. Li, Y. Zhao, S. Luo, *Nanotechnology* **2020**, *31*, 155402.

[8] C. Li, H. Su, K. Zhang, Z. Liu, H. Wang, D. Li, *J. Alloys Compd.* **2021**, *858*, 157714.

[9] D. Liu, C. Wang, K.-J. Jeong, J. Lee, *J. Alloys Compd.* **2020**, *832*, 152824.

[10] T. Li, H. Li, A. Qin, D. Zhang, F. Xu, *J. Power Sources* **2021**, *489*, 229510.

[11] J.-L. Xu, X. Zhang, Y.-X. Miao, M.-X. Wen, W.-J. Yan, P. Lu, Z.-R. Wang, Q. Sun, *Appl. Surf. Sci.* **2021**, *546*, 149163.

[12] J. Song, A. Y. Maulana, H. Kim, B. Yun, H. Gim, Y. Jeong, N. An, C. M. Futalan, J. Kim, *J. Alloys Compd.* **2022**, *921*, 166082.

[13] R. Li, L. Qing, W. Zhao, Y. k. Wu, J. Chen, W. Su, *Ionics* **2022**, *28*, 2613.

[14] W. Feng, V. S. Avvaru, R. R. Maca, S. J. Hinder, M. C. Rodriguez, V. Etacheri, *ACS Appl. Mater. Interfaces* **2021**, *13*, 27999.

[15] H. Kong, Y. Wu, W. Hong, C. Yan, Y. Zhao, G. Chen, *Energy Storage Mater.* **2020**, *24*, 610.

[16] L. Li, Q. Wang, X. Zhang, L. Fang, X. Li, W. Zhang, *Appl. Surf. Sci.* **2020**, *508*, 145295.

[17] Z. Zhang, Y. Huang, X. Liu, X. Wang, P. Liu, *Electrochim. Acta* **2020**, *342*, 136104.

[18] J. Zhao, C. Zhou, Y. Li, K. Cheng, K. Zhu, K. Ye, J. Yan, D. Cao, Y. Xie, G. Wang, *Int. J. Energy Res.* **2020**, *44*, 3883.

[19] M. Wang, Y. Huang, Y. Zhu, N. Zhang, J. Zhang, X. Qin, H. Zhang, *Electrochim. Acta* **2020**, *335*, 135694.

[20] J. Singh, S. Lee, P. Yadav, S. Kim, J. Kim, A. K. Rai, *ACS Appl. Energy Mater.* **2021**, *5*, 126.

[21] L. Wu, J. O. Yang, S. Guo, L. Yao, H. Li, S. Zhang, H. Yue, K. Cai, C. Zhang, C. Yang, Y. Cao, *Adv. Funct. Mater.* **2020**, *30*, 2001718.

[22] G. Fang, J. Zhou, Y. Cai, S. Liu, X. Tan, A. Pan, S. Liang, *J. Mater. Chem. A* **2017**, *5*, 13983.
